# Application of high field magnetic resonance microimaging in polymer gel dosimetry

**DOI:** 10.1002/mp.14186

**Published:** 2020-05-15

**Authors:** Agnieszka Skorupa, Aleksandra Woźnica, Mateusz Ciszek, Michał Staniszewski, Marek Kijonka, Marek Kozicki, Bożena Woźniak, Andrzej Orlef, Andrzej Polański, Łukasz Boguszewicz, Maria Sokół

**Affiliations:** ^1^ Department of Medical Physics Maria Skłodowska‐Curie National Research Institute of Oncology Gliwice Branch Wybrzeże Armii Krajowej 15 Gliwice 44-101 Poland; ^2^ Institute of Informatics Silesian University of Technology Akademicka 16 Gliwice 44-100 Poland; ^3^ Department of Mechanical Engineering, Informatics and Chemistry of Polymer Materials Lodz University of Technology Żeromskiego 116, A33 Lodz 90-924 Poland; ^4^ GeVero Co. Tansmana 2/11 Lodz 92-548 Poland

**Keywords:** 3D polymer gel dosimetry, high‐field MRI, microimaging, VIP, VIPAR^nd^

## Abstract

**Purpose:**

The purpose of this work was to examine the suitability of VIPAR^nd^ polymer gel–9.4 T magnetic resonance microimaging system for high spatial resolution dose distribution measurements.

**Methods:**

The VIPAR^nd^ samples (3 cm in outside diameter and 12 cm in height) were exposed to ionizing radiation by using a linear accelerator (Varian TrueBeam, USA; 6 MV x‐ray beam). In the calibration stage, nine gel dosimeter vials were irradiated in a water phantom homogenously to the doses from 1.5 to 30 Gy in order to obtain R2‒dose relation. In the verification stage, two gel dosimeter vials were irradiated in the half beam penumbra area of 10 × 10 cm radiation field using the amount of monitor units appropriate to deliver 20 Gy at the field center. The gels were imaged on a vertical 9.4 T magnetic resonance (MR) microimaging scanner using single slice and multislice (9 slices) multiecho (90 × 7 ms) sequences at the spatial resolutions of 0.2–0.4 × 0.2–0.4 × 3 mm^3^ and 0.2–0.4 × 0.2–0.4 × 1 mm^3^ respectively. The gels were subjected to microimaging during the period of two weeks after irradiation. The reference data consisted of the dose profiles measured using the diode dosimetry, radiochromic film, ionization chamber, and the water phantom system.

**Results:**

The VIPAR^nd^‒9.4 T MR microimaging system was characterized by the dose sensitivity of 0.067 ± 0.002 Gy^−1^ s^−1^ at day 3 after irradiation. The dose resolution at 10 Gy (at *P* = 95%) was equal to 0.42 Gy at day 3 after irradiation using a single slice sequence (0.2 × 0.2 × 3 mm^3^) and 2.0 Gy at day 4 after irradiation using a multislice sequence (0.2 × 0.2 × 1 mm^3^) for one signal acquisition (measurement time: 15 min). These values were improved by ~1.4‐fold when using four signal acquisitions in the single slice sequence, and by ~2.78‐fold for 12 signal acquisitions in the multislice sequence. Furthermore, decreasing the in‐plane resolution from 0.2 × 0.2 mm^2^ to 0.4 × 0.4 mm^2^ resulted in a dose resolution of 0.3 Gy and 1 Gy at 10 Gy (at *P* = 95%) for one signal acquisition in the single slice and multislice sequences respectively (measurement time: 7.5 min).

As reveals from the gamma index analysis the dose distributions measured at days 3–4 postirradiation using both VIPAR^nd^ verification phantoms agree with the data obtained using a silicon diode, assuming 1 mm/5% criterion. A good interphantom reproducibility of the polymer gel dosimetry was proved by monitoring of two phantoms up to 10 days after irradiation. However, the agreement between the dose distributions measured using the diode and polymer gel started to get worse from day 5 after irradiation.

**Conclusion:**

The VIPAR^nd^–9.4T MR microimaging system allows to obtain dose resolution of 0.42 Gy at 10 Gy (at *P* = 95%) for a spatial resolution of 0.2 × 0.2 × 3 mm^3^ (acquisition time: 15 min). Further studies are required to improve a temporal stability of the gel‐derived dose distribution.

## 1 Introduction

Progress in radiotherapy treatment planning and delivery methods (stereotactic radiosurgery, CyberKnife, intensity modulated radiotherapy) enables high precision localized dose delivery to delineate the target volume *via* employment of the therapy fields sizes smaller than 3 cm. The dosimetric characterization of such fields and verification of the treatment plans is difficult with the application of conventional detectors (such as ionization chambers) due to the lack of electronic equilibrium and the volume averaging effects in the areas of high dose gradients. Although microchambers, diodes, diamond detectors, and radiographic/radiochromic films are typically used for small field dosimetry, there is not a “gold standard” single detector fulfilling the requirements of high spatial resolution, tissue equivalence, low energy, and directional dependence. Therefore, a combination of various detectors is advised in clinical dosimetry.^1^

Three‐dimensional (3D) polymer gel dosimetry has been used in radiotherapy for 25 yr and the new methodologies are being developed.^2^ The principle behind the method is a radical polymerization and the crosslinking reactions of the vinyl monomers initiated by the water radiolysis products after a gel dosimeter irradiation. The degree of polymerization and crosslinking depends on the absorbed dose and can be quantified by using a magnetic resonance imaging (MRI) scanner by spin‐spin relaxation rate (R2) mapping. The clinical implementation of a polymer gel as a standard dosimeter is difficult due to the complexity of the overall process (fabrication, storage, irradiation, stabilization, imaging). However, according to Schreiner,^3^ the current knowledge related to 3D chemical dosimetry allows it to be used for commissioning the treatment planning system and benchmarking performance (both treatment planning and dose delivery), as well as for a periodic and routine patient specific treatment quality assurance. Presently, a routine application of 3D dosimetry might be more feasible owing to some commercial products available (see Table III in Schreiner^3^) and a number of successful application studies published.^4^, ^5^, ^6^ This 3D dosimetry technique may be attractive for small field dosimetry as well,^7^, ^8^, ^9^, ^10^, ^11^ and where the need for higher spatial resolution readout is more evident that for MV small fields. These include brachytherapy, beta‐emitting eye plaques, nano‐particle enhanced dose delivery, microbeam RT, and interface dosimetry for low‐ and medium‐energy photon fields. The opportunity of three‐dimensional high resolution dose read‐out and tissue equivalence, should be listed among the main advantages of the method. Of note, the dosimetric polymer gels do not disturb small beams, address the positioning challenges, and minimize the volume averaging effects.

To date, the clinical 1.5 or 3 T MRI scanners have been frequently used for conversion of the R2 maps to the absorbed dose maps after application of an R2‐dose calibration relation.^10^, ^11^ Although the voxel volume was around several mm^3^ in the vast majority of works, the feasibility of dose visualization at a submillimeter in‐plane resolution scale was also presented.^7^, ^12^ Bayreder et al., using a modulation transfer function approach, proved that a polymer gel dosimetry is possible at a resolution of 0.094 × 0.094 × 1 mm^3^ with a 3 T scanner equipped additionally with a special gradient system and a microimaging coil.^12^ The importance of extending the investigations to higher magnetic field strengths (above 3 T) was underscored by Hassani et al.^7^ The dose distribution imaging at increased spatial resolution achievable at these fields could open new possibilities for small beam dosimetry. However, apart of several published works,^8^, ^9^ application of high magnetic field microimaging in polymer gel dosimetry remains an unexplored area of research.

*N*‐vinylpyrrolidone based polymer gel dosimeters have been found to be useful in small field dosimetry.^10^, ^11^ Since their introduction in 1999,^13^ its chemical composition has been modified by several groups in order to lower the threshold dose, increase the dose sensitivity, or to replace a natural gelatin matrix by a synthetic one.^4^, ^14^, ^15^, ^16^, ^17^ A review of the chemical modifications of *N‐*vinylpyrrolidone based polymer gel dosimeters is presented elsewhere.^17^ High dose sensitivity is an important feature to fulfill strict dose resolution requirements imposed on the dosimeters used in radiotherapy. The minimal detectable dose difference depends both on a chemical formula and on the read‐out system.

In this work, the dosimetric performance of the VIPAR^nd^ polymer gel (VIPAR^nd^ or VIP abbreviation for this dosimeter is used interchangeably^18^) coupled with a 9.4 T MR micro‐imaging system was assessed. The main advantage of this formula is a wide range of the linear R2‐dose response (0.5–35 Gy), covering the doses used clinically in stereotactic radiosurgery. However, the dose sensitivity of VIPAR^nd^ is relatively low (ca. 0.0888 Gy^−1^s^−1^ at 1.5 T).^18^ To conceptualize the precision of signal acquisition required to fulfill the 2% dose resolution criterion imposed on the detectors used in radiotherapy (recommended by ICRU Report No. 42^19^), the R2 corresponding to 10 Gy (in the order of 4 s^−1^ at 1.5 T) should be measured with an overall error lower than 9% (at a confidence level *P* of 52%). Taking into account that polymer gel dosimetry is a multistage process, being prone to several sources of error, minimization of the noise in the R2 maps using a high magnetic field microimaging seems to be important for an effective use of the VIPAR^nd^ gel dosimeter for high spatial resolution dosimetry.

To be successfully used for small field dosimetry the Vipar^nd^ — 9.4 T MRI microimaging system should be first evaluated in a simple and well‐defined dose radiation gradient. In this work the polymer gel was placed in the half‐beam penumbra region of the 10 cm × 10 cm field. The obtained dose distribution was compared to the reference data consisting of the results of the diode and radiochromic film measurements taken as an approximation of the true profiles. A successful application of the Vipar^nd^ — 9.4 T MRI microimaging system for a high spatial resolution verification of a well‐defined dose distribution could open new possibilities for small field dosimetry.

## 2 Materials and Methods

### 2.A. Gel dosimeter preparation

The VIPAR^nd^ gel dosimeter was manufactured according to the methodology by Kozicki et al.^18^ The polymer solution was transferred into 10 cylindrical poly(methyl methacrylate) vials (GeVero Co., Poland) of 3 cm in outside diameter and 12 cm in height (the diameter of the dosimeter inside the vial is about 2.7 cm and its length available for irradiation is about 10 cm). The vials were designed in such way that they fit closely to the measurement cell of the MRI instrument. It should be noted, that the vials were equipped with a pressure compensating valve that protects the gel dosimeter inside from cracking and formation of empty spaces due to the gel dosimeter shrinkage during solidification. This propagates onto the quality of images obtained by using different 3D scanning techniques. All VIPAR^nd^ gel dosimeter phantoms were prepared at the Lodz University of Technology, Poland. The time between the manufacturing and irradiation of the dosimeters amounted to 3 days.

### 2.B. Gel dosimeter irradiation

The VIPAR^nd^ samples were transferred to the Maria Skłodowska‐Curie National Research Institute of Oncology, Gliwice Branch, Poland by a courier company for the irradiation and MRI scanning. Although the samples were protected against unexpected temperature variation and mechanical damage, the exact temperature history of the samples during the transfer was unknown.

The samples were exposed to ionizing radiation using a linear accelerator (Varian TrueBeam, USA). The 6 MV x‐ray beam at 300 JM/min directed perpendicularly to a water surface was used. The beam output was 1 cGy/JM in the reference conditions (z_ref_ = 5 gcm^−2^, SCD = 100 cm, 10 × 10 cm^2^). The samples were placed in a water phantom (MP3 ‐ M Therapy Beam Analyzer, PTW, Freiburg, Germany) at a depth of 6 cm with the vial long axis centered perpendicularly to the beam axis. To prevent dose gradient along the radiation beam axis, all vials were irradiated twice, before and after 180^o^ rotation around the vial long axis.

The experiment was divided into two stages: (a) calibration and (b) verification. In the calibration stage, nine gel dosimeter vials were irradiated homogenously using two opposing beams to the doses of 1.5, 3, 5, 8, 10, 14, 20, 25, and 30 Gy in order to obtain the R2‐dose relation (R2 denotes the reciprocal of T2, the MR spin‐spin relaxation time). Each gel dosimeter vial was exposed to 20 × 20 cm^2^ radiation field size with the number of monitor units adequate to obtain the planned doses. The dose values were estimated on the basis of the dosimetric measurements using a Semiflex 3D Chamber and Unidos E (both PTW‐Freiburg, Germany) for the same water phantom. One gel dosimeter vial was left nonirradiated. In the verification stage, two gel dosimeter vials were irradiated in the half beam penumbra area to investigate accuracy of the high gradient dose distribution measurement. The vial long axis was centered in a half beam penumbra area. The dose of 20 Gy was prescribed at a point shifted 5 cm from the field central axis in Y direction, at the depth of 6 cm. The field size was: X: 10 cm, Y: 10 cm at SSD 100 cm, with a fully closed Y2 jaw and open Y1 jaw.

The reference data consisted of the absolute dose profiles measured using Dosimetry Diode E (type 60017), Semiflex 3D Chamber (type 31021) and MP3 ‐ M Therapy Beam Analyzer (all PTW‐Freiburg, Germany) and the film dosimetry system.

The radiochromic film dosimetry was performed with a GAFCHROMIC EBT3 film (International Specialty Products, USA, lot number06081601, sheet dimensions of 20.3 × 25.4 cm^2^). The film irradiation was performed using the set‐up mimicking the set‐up used for gel dosimeters, with the sheets of solid water instead of a water tank. To obtain a calibration curve the film samples were cut into 2 × 5 cm^2^ pieces and irradiated using 6 MV, perpendicularly oriented beam from TrueBeam (Varian, USA) accelerator. Films were exposed in a water equivalent RW3 slab phantom (PTW, Freiburg, Germany) consisting of 30 × 30 cm^2^ sheets. The samples were placed at the isocenter of the accelerator with 5 cm of the phantom material over and 10 cm under the film. The source‐to‐film distance was 100 cm. A 10 × 10 cm^2^ field size defined at the isocenter was used. The calibration curve was obtained for the following dose levels: 0, 0.2, 0.5, 1, 2, 3, 4, 5, 6, 7, 9, 10 Gy. The Epson Perfection V850 Pro scanner (Seiko Epson Corporation, Japan) working in a transmission mode was used to scan the film. The images were acquired in a 48‐bit RGB scanning mode at the resolution of 72 dpi. The film pieces were placed in the center of the scanner bed to mitigate scanner nonuniformity effect. The raw dose images were imported into the RIT 113 (Radiological Imaging Technology, USA) analysis software. The mean pixel value for 1 cm × 1 cm^2^ ROI of each calibration film piece was assigned to the corresponding delivered dose to obtain the film dose response curve. The red color channel of the images was used.

For a verification stage the film sheet was cut into four 5 × 13 cm^2^ samples. To obtain the dose distribution profile across the examined penumbra area the films were placed in the RW3 slab phantom under the geometric and radiation beam conditions corresponding to those used for the verification gel vials irradiation in a water phantom. The films were irradiated to the dose of 8 Gy at the prescription point. RIT 113 analysis software was used to obtain the film dosimetry reference data by averaging the adjacent line profiles for noise reduction.

### 2.C. MR microimaging

Before the dose read‐out, all gel dosimeters were stored in an MR room to equilibrate them to room temperature (21°C). The measurements started 24 h postirradiation and were continued up to 14 days. The microimaging experiments were performed on a vertical 9.4 T Bruker scanner (Germany) equipped with a Micro2.5 gradient system and a transmit/receive birdcage radio frequency coil with an inner diameter of 30 mm. The gel dosimeter vials were positioned with their long axes parallel to the direction of the B0 field. The parameters of the basic sequence used in this work were as follows: repetition time (TR) 6000 ms, number of echoes 90, echo time (TE) from 7 to 630 ms, echo spacing 7 ms, in‐plane resolution of 0.2 × 0.2 mm^2^. Both single slice (3 mm thickness) and the multislice (nine slices acquired with use of an interleaved mode, 1 mm thickness) versions of this sequence were applied. The measurement time for one signal acquisition was equal to 15 min for both sequence types.

For each pixel the spin‐spin relaxation rate (R2) was calculated by a mono‐exponential fitting according to the equation:

(1) $$S_{i}=kS_{0}exp\left(-R2\cdot i\Delta TE\right)$$

where S_i_ denotes the signal intensity obtained for i‐th TE value, k is a proportionality constant related to signal gain or attenuation and S_o _is the proton density. The echoes falling within the range from 7‒28 ms were excluded from the fitting due to the imperfections of the spin‐spin relaxation decay curve, by analogy to the observations published earlier.^20^

The information about the signal to noise ratios (SNRs) of the obtained images and the relationship between the relative R2 uncertainty and a number of echoes included in a mono‐exponential fitting is provided in supplementary file 1 and supplementary file 2.

#### 2.C.1. Microimaging of calibration vials

Although VIPAR^nd^ gel dosimeter was used for relative dosimetry in this work, a considerable attention was paid to microimaging of homogenously irradiated calibration vials under various acquisition parameters and at various time periods after irradiation. These measurements were conducted according to the following schedule:

- Determination of the dose resolution of the VIPAR^nd^–9.4 T MR microimaging system (days 1‒2 after irradiation) for different number of the signal averages.

The calibration gel vials, irradiated to 1.5, 10, and 20 Gy, were imaged using a basic single slice and a multislice multiecho sequence (the acquisition parameters are mentioned above) for a different number of the signal averages: 1, 4, 8 for a single slice and 1, 4, 8, and 12 for a multislice technique.

The dose resolution ( $D_{\Delta }^{p}$ , a minimal detectable dose difference for a given confidence level, p) was computed according to the equation:

(2) $$D_{\Delta }^{p}=k_{p}\sqrt{2}\frac{\sigma_{R2}}{\alpha }$$

where k_p_ denotes a coverage factor (1.96 for *P* = 95%, 1 for *P* = 68 % and $1/\sqrt{2}$ for *P* = 52%), α is a dose sensitivity and σ_R2_ is a mean standard uncertainty in the circular region of interest (ROI) positioned in the center of the phantom.^21^ The diameter of the ROI was equal to 3 mm corresponding to the area of 7.065 mm^2^ (~192 pixels).

The dose sensitivity α was estimated from a linear fitting of the R2‐dose relationship:

(3) $$R2=R2_{0}+\alpha D$$

where R2 denotes a mean relaxation rate in the region of interest positioned in the center of the phantom corresponding to irradiation dose D, R2_0_ is an offset of the R2‐dose relation.

- Assessment of the temporal stability of the R2‒dose relationship and dose resolution (days 3‒14 postirradiation).

The measurements of the R2‐dose response curves (involving all irradiated gel dosimeter vials) were conducted at 3, 5, 7, 10, and 14 days postirradiation for a basic single slice sequence and at 4, 5, 7, 10, and 14 days for a multislice technique. Since the R2 values were averaged over the regions of interest containing ~192 pixels, one signal acquisition was used. From these measurements the temporal changes in the calibration curves and dose resolution were obtained.

- Determination of the R2‒dose curves and dose resolution for a variable voxel size in a single slice technique (day 3 after irradiation).

The calibration gel vials were imaged using a basic single slice sequence (voxel resolution: 0.2 × 0.2 × 3 mm^3^, one signal average) and the sequences modified with respect to the voxel size. The R2 mapping was performed for an in‐plane resolutions of 0.2 × 0.2 mm^2^ (acquisition time: 15 min), 0.3 × 0.3 mm^2^ (acquisition time: 10 min), and 0.4 × 0.4 mm^2^ (acquisition time: 7.5 min) under a fixed slice thickness of 3 mm. The effect of variable slice thickness (1, 2, and 3 mm) under a fixed in‐plane resolution of 0.2 × 0.2 mm^2^ was also examined. The R2 values were averaged over the regions of interest containing ~192 pixels for a resolution of 0.2 × 0.2 mm^2^, ~86 pixels for a resolution of 0.3 × 0.3 mm^2^, ~52 pixels for a resolution of 0.4 × 0.4 mm^2^.

- Determination of the R2‐dose curves and a dose resolution for a variable voxel size in a multislice technique (day 4 after irradiation).

The calibration gel vials were imaged using a basic multislice multiecho sequence (voxel resolution: 0.2 × 0.2 × 1 mm^3^) and the sequences modified with respect to the voxel size. The R2 mapping was performed for the in‐plane resolutions of 0.2 × 0.2 mm^2^ (acquisition time: 15 min), 0.3 × 0.3 mm^2^ (acquisition time: 10 min), and 0.4 × 0.4 mm^2^ (acquisition time: 7.5 min) under a fixed slice thickness of 1 mm. Similar measurements were performed for a fixed slice thickness of 2 mm. The effect of a variable slice thickness (1, 2 and 3 mm) under a fixed in plane resolution of 0.2 × 0.2 mm^2^ was also evaluated. R2 values were averaged over the regions of interest containing ~192 pixels for a resolution of 0.2 × 0.2 mm^2^, ~86 pixels for a resolution of 0.3 × 0.3 mm^2^, ~52 pixels for a resolution of 0.4 × 0.4 mm^2^.

- Determination of the effect of the number of echoes and echo spacing on a dose resolution (day 6 after irradiation).

The gel vials irradiated with 1.5, 5, 10, 14, and 20 Gy were imaged using a basic multislice sequence (7 ms × 90, 0.2 × 0.2 × 1 mm^3^, one signal average) and two sequences modified with respect to the number of echoes and echo spacing (20 ms × 32, 10 ms × 64). The effect on dose resolution was evaluated.

#### 2.C.2. Microimaging of verification vials

**Fig. 1:**
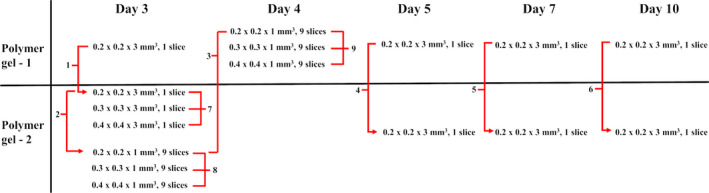
The scheme presenting the time periods of the 9.4 T MR microimaging of the VIPAR^nd^ verification vials. The numbers 1–9 represent comparisons between various imaging sequences and phantoms. [Color figure can be viewed at wileyonlinelibrary.com]

The dose distributions in the VIPAR^nd^ verification vials were read out along with the measurements of the calibration vials for the assessment of temporal and spatial stability. The scheme presenting the time periods of the R2 mapping is shown in Fig. 1. The single slice measurements (0.2 × 0.2 × 3 mm^3^) were performed at days 3, 5, 7, and 10 for both vials, while the multislice imaging (0.2–0.4 × 0.2–0.4 × 1 mm^3^, 9 slices) was done for the phantom 2 (at day 3) and for the phantom 1 (at day 4). The phantom 2 was imaged at various in‐plane resolutions (0.2‒0.4 × 0.2‒0.4 mm^2^) using a single slice technique at day 3.

**Fig. 2:**
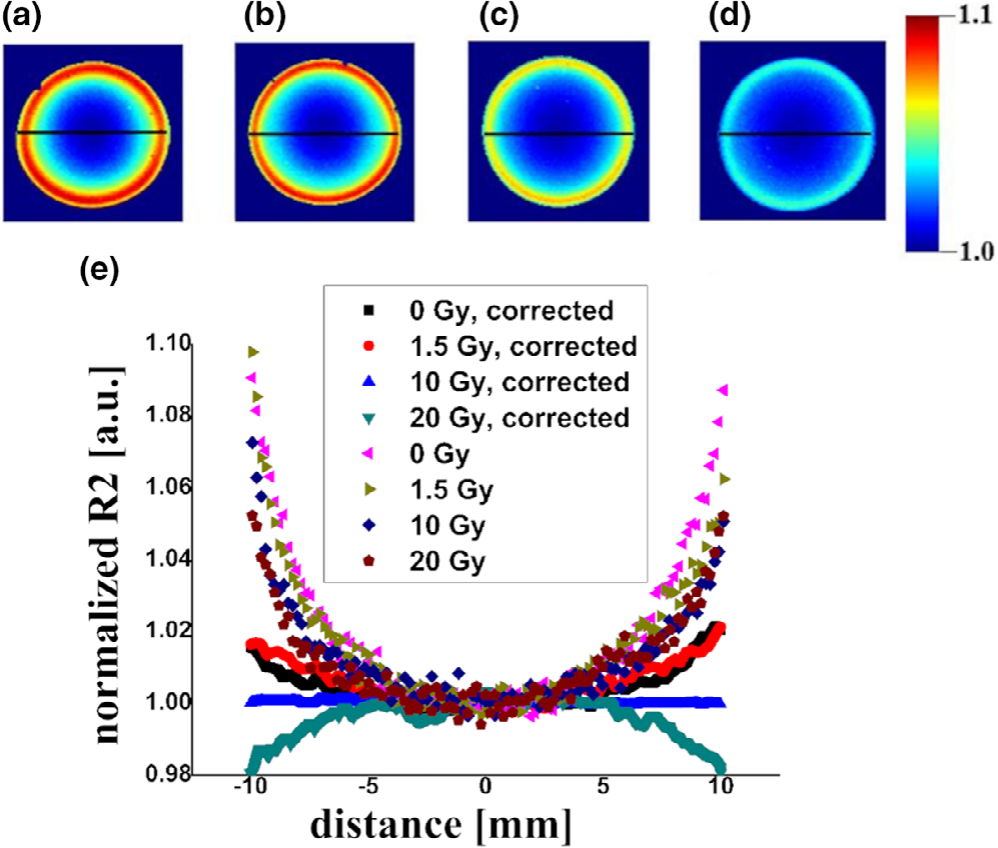
The R2 maps are obtained from the nonirradiated phantom (a), calibration vials irradiated homogenously to the doses of 1.5 Gy (b), 10 Gy (c) and 20 Gy (d) normalized to the mean R2 in the region of interest located in the vial center (single slice technique, 0.2 × 0.2 × 3 mm^3^, one signal average). The normalized R2 (a. u.) as a function of position “before” and “after” correction for nonuniformity in the vials irradiated with 0, 1.5, 10, and 20 Gy measured along the black lines (d). [Color figure can be viewed at wileyonlinelibrary.com]

The R2 maps computed for the verification phantoms were corrected for a signal nonuniformity (manifested by an increase of the R2 values near the edges of the coil) using the R2 maps obtained for the homogenously irradiated vials as templates according to the *response matrix method* proposed by Lepage et al.^22^ Of note, the images of homogenously irradiated vials were measured at approximately the same time period (within 24 h) as the R2 images for the verification phantoms. The exemplary unsmoothed templates corresponding to the doses of 0, 1.5, 10, and 20 Gy and (acquired at day 3 postirradiation using a single slice technique at a resolution of 0.2 × 0.2 × 3 mm^3^, one signal average) are presented in Figs. 2(a)–2(d). These templates were calculated as the R2 maps normalized to the mean R2 in the circular region of interest (diameter 3 mm) located in the phantom center. They were subjected to median filtering before calculation of the correction factors. It is apparent that the image inhomogeneity is dependent on R2, as previously shown.^22^ Thus, the correction factors applied to each pixel of the R2 maps in the verification phantoms were dependent both on the position within the coil and on the actual R2. All computations were performed in Matlab (v. 2016b, MathWorks, USA).

Figure 2(e) shows that residual variation due to the image nonuniformity amounts to ±2% at the distance of ±10 mm around the beam axis for the dose range from 0 to 20 Gy.

**Fig. 3:**
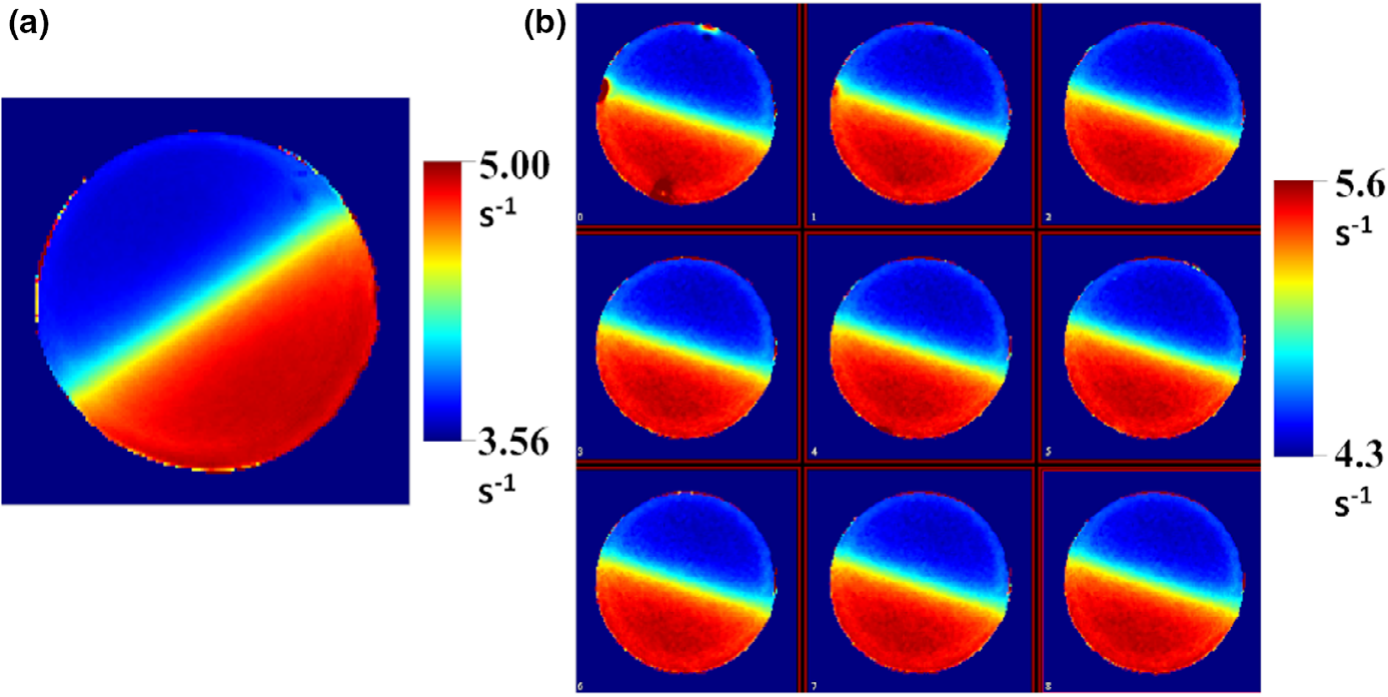
The inhomogeneity corrected R2 maps obtained for a verification phantom 1 using single slice (0.2 × 0.2 × 3 mm^3^, 4 averages, day 3) (a) and multislice sequences (0.2 × 0.2 × 1 mm^3^, 9 slices, 12 averages, day 4) (b). [Color figure can be viewed at wileyonlinelibrary.com]

The examples of the inhomogeneity corrected R2 maps obtained for a verification phantom 1 using the single (0.2 × 0.2 × 3 mm^3^, 4 averages, day 3) and multislice sequences (0.2 × 0.2 × 1 mm^3^, 12 averages, day 4) are presented in Fig. 3. These maps were converted to the R2_net_ (net irradiation effect on R2) maps by subtracting R2_0_ corresponding to the intercept of the dose‐response curve measured at approximately the same time periods as for the verification samples.

The dose distributions were extracted automatically. The beam central axis was coincident with the phantom center. Assuming that the dose distribution is one‐dimensional, the direction of the minimal standard deviation of R2 was found. The profiles were read out along the line perpendicular to this direction. The relative dose distributions were obtained by normalization of the R2_net_ values to 100 % at a distance of 6.3 mm from the beam axis (phantom center).

The verification experiment was designed to allow a direct comparison of (Fig. 1):

− the dose distributions between two phantoms at a particular time point (single slice technique: comparison 1 (at day 3), comparison 4 (at day 5), comparison 5 (at day 7), comparison 6 (at day 10); multislice technique: comparison 3)),
− the data measured using the single slice and multislice techniques (comparison 1),
− the dose distributions measured at various in‐plane resolutions (single slice: comparison 7; multislice: comparisons 8 and 9).

The gamma index analysis^23^ was used to characterize a degree of similarity of the dose distributions obtained using the VIPAR^nd^–9.4 T MR microimaging system and the conventional detectors (diode and ionization chamber) in terms of the dose difference and distance to agreement. During these analyses various dose difference criteria (1‒6%) were checked for a fixed spatial tolerance of 1 or 0.5 mm. The profiles were considered as similar for the gamma index values below 1.

## 3 Results

### 3.A. Determination of the dose resolution of the VIPAR^nd^–9.4 T MR microimaging system (days 1‒2 after irradiation) for different number of signal averages

**Fig. 4:**
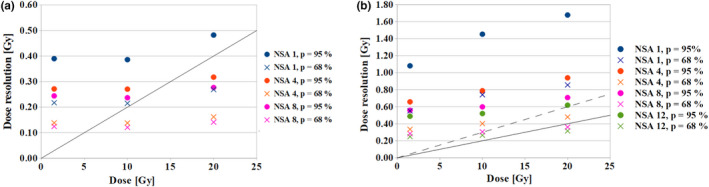
The dose resolution (at *P* = 95% and 68%) obtained using the basic single slice (0.2 × 0.2 × 3 mm^3^) (a) and basic multislice (0.2 × 0.2 × 1 mm^3^) (b) sequences for different number of signal averages. The solid and dashed lines indicate 2% and 3% dose resolution criteria respectively. NSA – number of signal averages. [Color figure can be viewed at wileyonlinelibrary.com]

In Fig. 4(a) the dose resolution obtained using a basic single slice sequence for different number of signal averages is presented. The minimal detectable dose difference (at *P* = 95%) is from 0.4 to 0.5 Gy for one signal average and can be improved to the value of ca. 0.3 Gy for the whole evaluated dose range using four signal averages (1.43‐fold improvement in the dose resolution at 10 Gy). It can be observed that for 4‒8 averages the 2% criterion of dose resolution (recommended by ICRU Report No. 42) can be fulfilled for the doses above ~13 Gy at 95% confidence level for the voxel size of 0.2 × 0.2 × 3 mm^3^. After reduction of the confidence level to 68% the dose resolution is below the 2% limit for the doses higher than ~7 Gy.

In Fig. 4(b) the dose resolution obtained using a basic multislice technique is shown to fall within the range from 1.1 to 1.7 Gy for one signal average, and can be improved to the value of ca. 0.5 Gy for 12 signal averages for the whole evaluated dose range (2.78‒fold improvement in the dose resolution at 10 Gy). The 2% limit of dose resolution cannot be fulfilled for the VIPAR^nd^ gel at the 95% confidence level using 12 signal acquisitions. However, 3% criterion can be met at 68% confidence level for the doses above 10 Gy for the voxel size of 0.2 × 0.2 × 1 mm^3^.

Based on the abovementioned results the number of signal averages was set to 4 for a single slice sequence and to 12 for a multislice technique used for microimaging of the verification phantoms.

### 3.B. Assessment of the temporal stability of the R2‐dose relationships and dose resolution (days 3‒14 postirradiation)

**Fig. 5:**
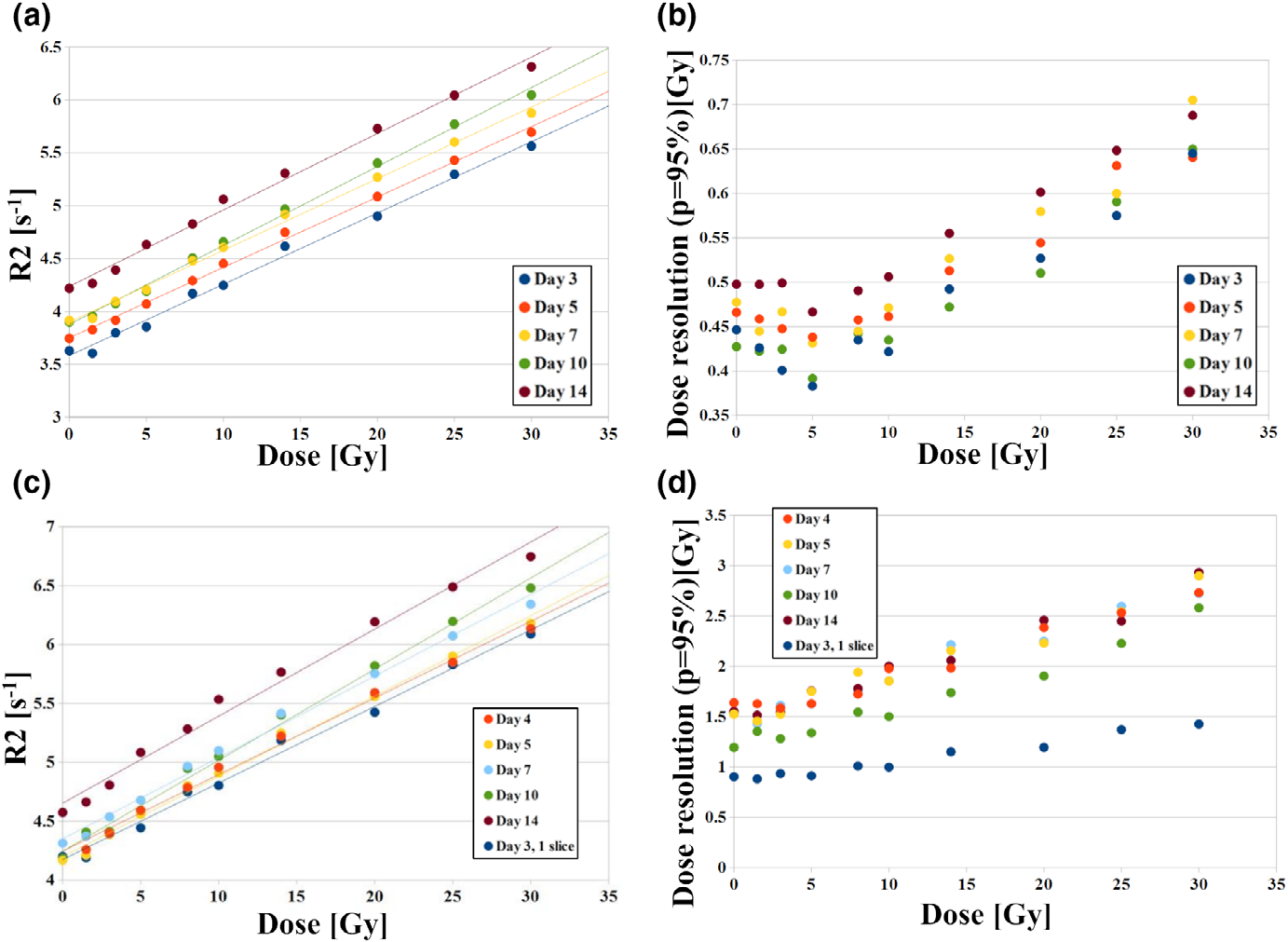
The temporal evolution of the R2‐dose calibration curves (a ‐ basic single slice sequence, c ‐ basic multislice sequence) and the dose resolution (b ‐ basic single slice sequence, d ‐ basic multislice sequence) over 14 days after irradiation for the VIPAR^nd^‒9.4 T MR system. Additionally, in Fig. 5(c) and 5(d) the R2‐dose relation and dose resolution are presented as obtained for a single slice sequence (0.2 × 0.2 × 1 mm^3^) at day 3 postirradiation. The absolute and relative R2 standard uncertainties are provided in Tables S4 and S5. [Color figure can be viewed at wileyonlinelibrary.com]

In Fig. 5 the temporal evolution of the R2‐dose calibration curves and dose resolution (at *P* = 95%) are shown for the single (0.2 × 0.2 × 3 mm^3^) and multislice techniques (0.2 × 0.2 × 1 mm^3^) obtained over 14 days after irradiation of VIPAR^nd^. In Tables I and II the slopes and offsets of the fitted lines and their uncertainties are presented. Both sequences yielded the dose sensitivity falling within a window from 0.065 to 0.077 Gy^−1^ s^−1^, while the R2_0_ values increased from 3.58 to 4.24 s^−1^ and from 4.17 to 4.35 s^−1^ for a single slice sequence and a multislice one, respectively, during the monitoring period. The dose resolution at 10 Gy was equal to 0.42 Gy at day 3 after irradiation using a single slice sequence and 2.0 Gy at day 4 using a multislice sequence. These parameters were quite stable during the monitoring period (the deviations below 0.1 Gy for the single slice measurements and 0.7 Gy for a multislice technique were detected). Although R2_0_ was gradually increased over time the R2 standard uncertainties remain fairly constant (Tables S4 and S5). Data shown in supplementary file 2 indicate that inclusion of 90 echoes in the mono‐exponential fitting of the relaxation decay is optimal both for the data acquired at day 3 and 14 after irradiation.

**Table I:** The dose sensitivity (α) and offset (R2_0_) obtained for VIPAR^nd^‒9.4 T MR using a basic single slice technique (0.2 × 0.2 × 3 mm^3^, NSA = 1) at different time periods after irradiation.

Time period after irradiation	Dose sensitivity (α) [Gy^−1^ s^−1^]	Offset (R2_0_) [s^−1^]
Day 3	0.067 ± 0.002	3.58 ± 0.03
Day 5	0.067 ± 0.001	3.75 ± 0.02
Day 7	0.068 ± 0.001	3.90 ± 0.02
Day 10	0.075 ± 0.001	3.88 ± 0.02
Day 14	0.072 ± 0.002	4.24 ± 0.03

**Table II:** The dose sensitivity (α) and offset (R2_0_) obtained for VIPAR^nd^‒9.4 T MR using a basic multislice technique (0.2 × 0.2 × 1 mm^3^, NSA = 1) at different time periods after irradiation. Additionally, the dose sensitivity and R2_0_ offset for a single slice sequence (0.2 × 0.2 × 1 mm^3^) at day 3 postirradiation are presented.

Time period after irradiation	Dose sensitivity [Gy^−1^ s^−1^]	Offset [s^−1^]
Day 4	0.065 ± 0.002	4.17 ± 0.04
Day 5	0.068 ± 0.002	4.25 ± 0.03
Day 7	0.069 ± 0.002	4.21 ± 0.03
Day 10	0.077 ± 0.002	4.35 ± 0.03
Day 14	0.074 ± 0.002	4.24 ± 0.03
Day 3, 1 slice	0.065 ± 0.002	4.65 ± 0.05

Additionally, the R2‒dose relations and dose resolution obtained using a single slice sequence (0.2 × 0.2 × 1 mm^3^) at day 3 postirradiation are presented in Figs. 5(c) and 5(d). The dose resolution at 10 Gy was equal to 1 Gy using this technique.

### 3.C. Determination of the R2‐dose curves and dose resolution for different voxel sizes in a single slice technique (day 3 after irradiation)

**Fig. 6:**
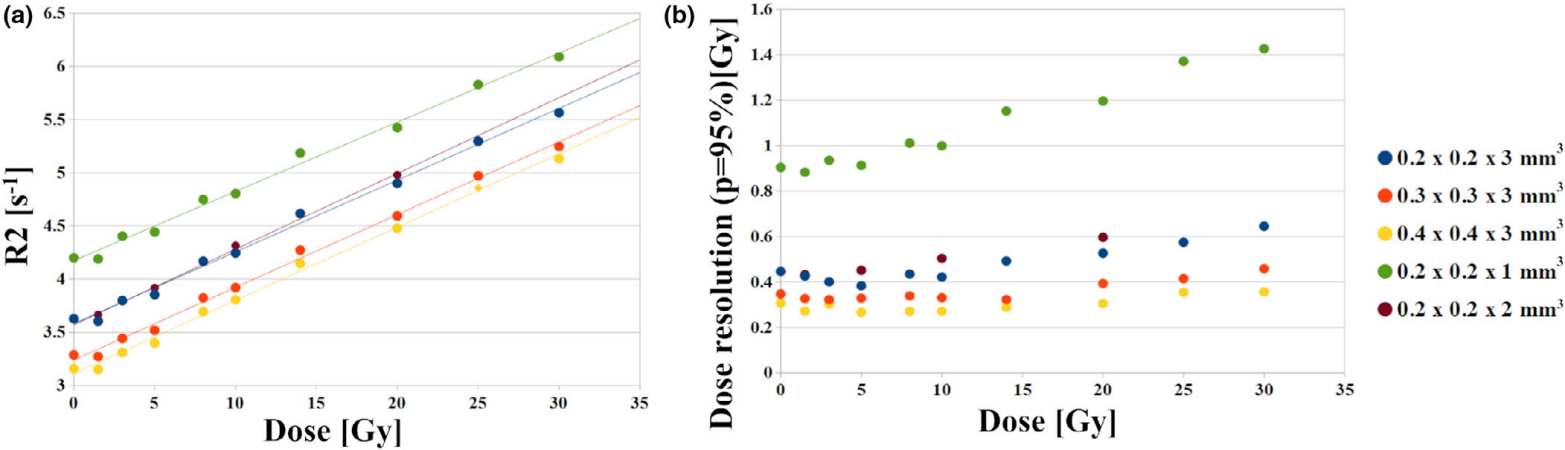
The VIPAR^nd^‒9.4 T microimaging R2‐dose relations (a) and dose resolution (b) for different in‐plane resolutions and slice thicknesses for a single slice sequence (day 3 after irradiation). The absolute and relative R2 standard uncertainties are provided in Table S6. [Color figure can be viewed at wileyonlinelibrary.com]

In Fig. 6(a) the R2‐dose relations for different in‐plane resolutions and slice thicknesses are presented for a single slice sequence while the slopes and intercepts of the fitted lines are collected in Table III. The changes in the voxel size affected mainly the intercept of the curves. The R2_0_ decreased from 3.58 to 3.11 s^−1^ with increasing pixel size from 0.2 × 0.2 mm^2^ to 0.4 × 0.4 mm^2^ under a fixed slice thickness of 3 mm. The slice thickness increase from 1 to 3 mm under a fixed in‐plane resolution of 0.2 × 0.2 mm^2^ led to a decrease of R2_0_ from 4.17 to 3.58 s^‐1^. Of note, the fitted parameters for a voxel size of 0.2 × 0.2 × 2 mm^3^ are very similar to those obtained for a voxel size of 0.2 × 0.2 × 3 mm^3^.

**Table III:** The dose sensitivity (α) and offset (R2_0_) obtained for VIPAR^nd^‒9.4 T MR by using a basic single slice technique for different voxel sizes.

Voxel size (mm^3^)	Dose sensitivity (α) [Gy^−1^s^−1^]	Offset (R2_0_) [s^−1^]
0.2 × 0.2 × 3	0.067 ± 0.002	3.58 ± 0.03
0.3 × 0.3 × 3	0.068 ± 0.002	3.24 ± 0.03
0.4 × 0.4 × 3	0.069 ± 0.002	3.11 ± 0.02
0.2 × 0.2 × 1	0.065 ± 0.002	4.17 ± 0.03
0.2 × 0.2 × 2	0.071 ± 0.002	3.75 ± 0.02

The dose resolution (*P* = 95%) at 10 Gy can be improved from 1 to 0.4 Gy by increasing slice thickness from 1 to 3 mm when using a fixed in‐plane resolution of 0.2 × 0.2 mm^2^, while the dose resolution (*P* = 95%) for the dose of ca. 10 Gy can be improved from 0.4 to 0.3 Gy by decreasing an in‐plane resolution from 0.2 × 0.2 mm^2^ to 0.4 × 0.4 mm^2^ under a fixed slice thickness of 3 mm [Fig. 6(b)].

### 3.D. Determination of the R2‐dose curves and dose resolution for different voxel sizes in a multislice technique (day 4 after irradiation)

**Fig. 7:**
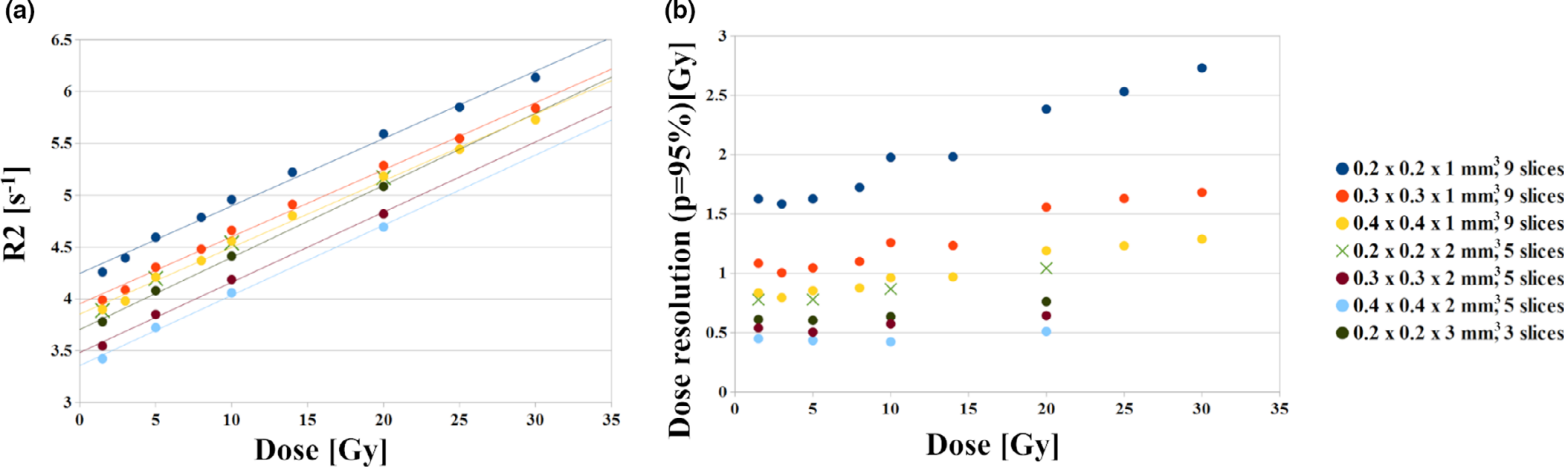
The VIPAR^nd^‒9.4 T MR R2‐dose relations (a) and dose resolution (b) for different in‐plane resolutions and slice thicknesses for a multislice sequence (at day 4 after irradiation). The absolute and relative R2 standard uncertainties are provided in Table S7. [Color figure can be viewed at wileyonlinelibrary.com]

The R2‐dose curves for different in‐plane resolutions and slice thicknesses for a multislice sequence are presented in Fig. 7(a). The slopes and intercepts of the curves are collected in Table IV. The R2_0_ falls from 4.27 to 3.91 s^−1^ with increasing pixel size from 0.2 × 0.2 mm^2^ to 0.4 × 0.4 mm^2^ under a slice thickness of 1 mm. Increasing the slice thickness from 1 to 3 mm for the in‐plane resolution of 0.2 × 0.2 mm^2^ led to the drop of the R2_0_ values from 4.27 s^−1^ to 3.36 s^−1^.

**Table IV:** The dose sensitivity (α) and offset (R2_0_) obtained for VIPAR^nd^‒9.4 T MR by using a basic multislice technique for different voxel sizes.

Voxel size (mm^3^)	Dose sensitivity (α) [Gy^−1^ s^−1^]	Offset (R2_0_) [s^−1^]
0.2 × 0.2 × 1	0.064 ± 0.002	4.27 ± 0.03
0.3 × 0.3 × 1	0.062 ± 0.003	4.01 ± 0.05
0.4 × 0.4 × 1	0.061 ± 0.003	3.91 ± 0.04
0.2 × 0.2 × 2	0.068 ± 0.003	3.83 ± 0.04
0.3 × 0.3 × 2	0.070 ± 0.002	3.70 ± 0.03
0.4 × 0.4 × 2	0.068 ± 0.003	3.48 ± 0.03
0.2 × 0.2 × 3	0.008 ± 0.003	3.36 ± 0.03

The dose resolution at 10 Gy could be improved from 2 Gy to 1 Gy by decreasing the in‐plane resolution from 0.2 × 0.2 × 1 mm^3^ to 0.4 × 0.4 × 1 mm^3^ [Fig. 7(b)], whereas increasing the slice thickness from 1 to 3 mm improves the dose resolution from 2 to 0.7 Gy at 10 Gy.

### 3.E. Determination of the effect of the number of echoes and echo spacing on dose resolution

**Fig. 8:**
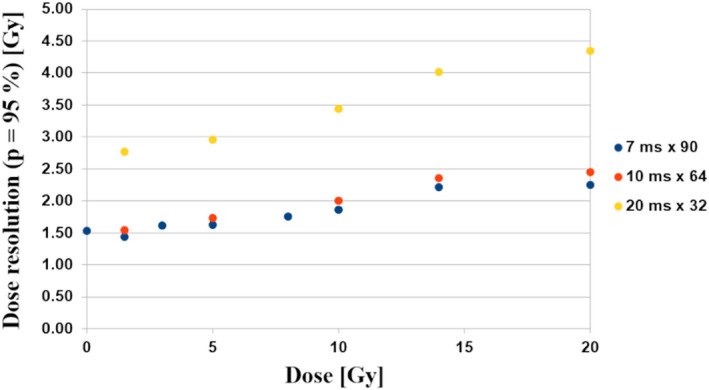
The dose resolution vs absorbed dose for VIPAR^nd^‒9.4 T MR for different number of echoes and a fixed total echo time of 630‒640 ms. [Color figure can be viewed at wileyonlinelibrary.com]

A relationship between the dose resolution and the absorbed dose for different number of echoes and a fixed total echo time of 630‒640 ms is shown in Fig. 8. As seen, changing the sampling of transverse relaxation decay from 20 ms × 32 to 7 ms × 90 results in an increase in the dose resolution at 10 Gy from 3.5 to 1.9 Gy.

### 3.F Microimaging of the verification phantoms

**Fig. 9:**
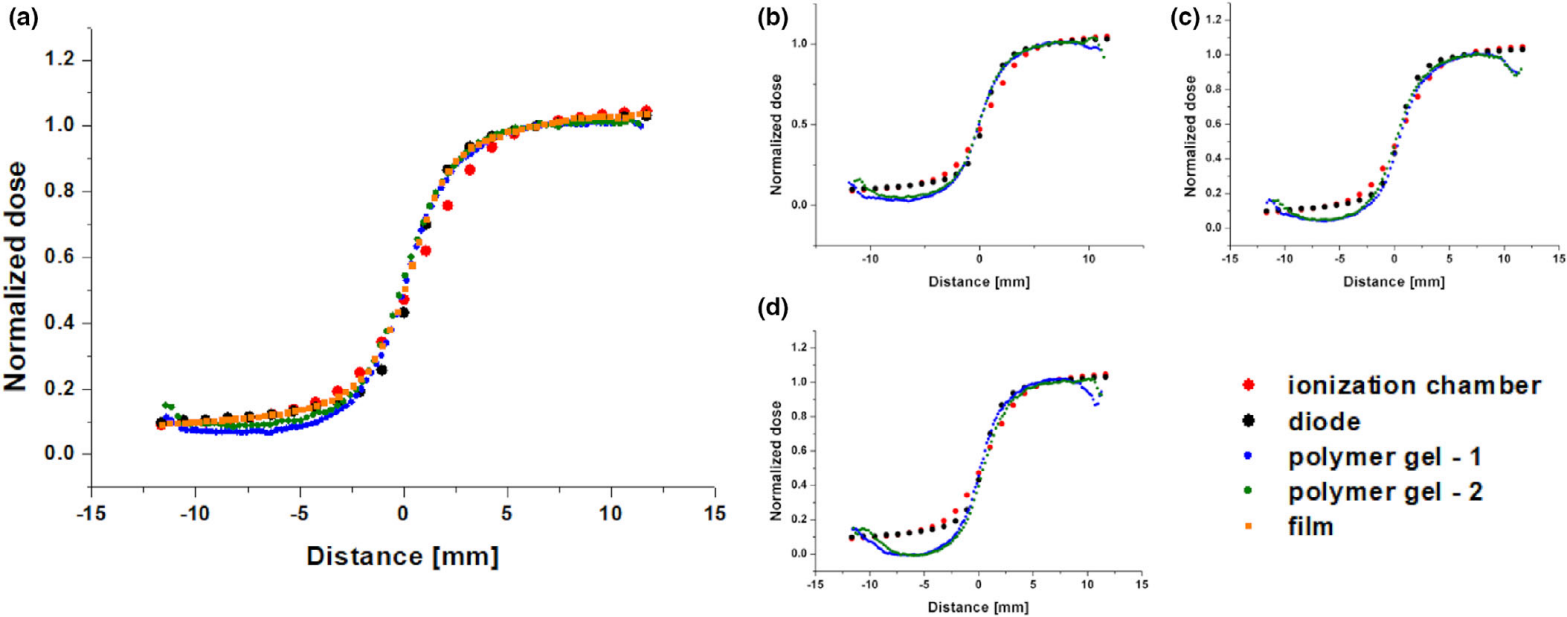
The relative dose profiles through the irradiated‐unirradiated transition zone obtained using a basic single slice microimaging of two VIPAR^nd^ verification gel vials at day 3(a), 5(b), 7(c), and 10(d) days after irradiation. The uncertainties of the normalized dose are provided in Tables [Link], [Link], [Link], [Link], while the uncertainties for the film data are shown in Table S12. [Color figure can be viewed at wileyonlinelibrary.com]

In Fig. 9(a) the relative dose profiles are presented that correspond to the irradiated‐unirradiated transition zone obtained using a basic single slice microimaging of two VIPAR^nd^ verification gel vials at day 3 postirradiation (comparison 1). These profiles are almost identical. Additionally, the corresponding data measured using a diode detector, radiochromic film and an ionization chamber are superimposed. The penumbra widths were found to be from 3.7 to 4.0 mm for the diode, polymer VIPAR^nd^ gel and film detectors, while the higher value was obtained for an ionization chamber (Table V). The gamma index analysis shows that the dose distributions measured using both VIPAR^nd^ verification phantoms agree with the data obtained using a silicon diode, assuming 1 mm/5% (or 0.5 mm/5%) criterion for the phantom 1 and 1 mm/2.5% (or 0.5 mm/4%) limit for the phantom 2. The distances of ±11.5 mm around the beam axis (point 0 mm, see Fig. 9) were taken into account in this analysis. A good interphantom reproducibility of the polymer gel dosimetry was proved by monitoring of both phantoms up to 10 days after irradiation [comparisons: 4, 5, and 6 presented in Figs. 9(b)–9(d) respectively]. However, starting from day 5 large discrepancies between the results obtained from a polymer gel and a silicone diode were observed in the low‐dose region (for the doses below 20%) and at the boundaries of the phantom (for the doses>80%).

**Table V:** The penumbra widths obtained using the VIPAR^nd^ polymer gel, diode, and ionization chamber measurements.

Detector	20–80% penumbra width (mm)
Polymer gel	3.90 ± 0.15
Film	4.19 ± 0.09
Silicone diode	3.70
Ionization chamber	5.80

The mean penumbra width and its standard deviation for a gel dosimetry were obtained from 10 adjacent profiles in two gel verification vials (measured using a single slice sequence at the resolution of 0.2* × *0.2* × *3 mm^3^ at day 3 postirradiation). The values for a film dosimetry were obtained from 10 adjacent profiles measured in four separate film sheets.

**Fig. 10:**
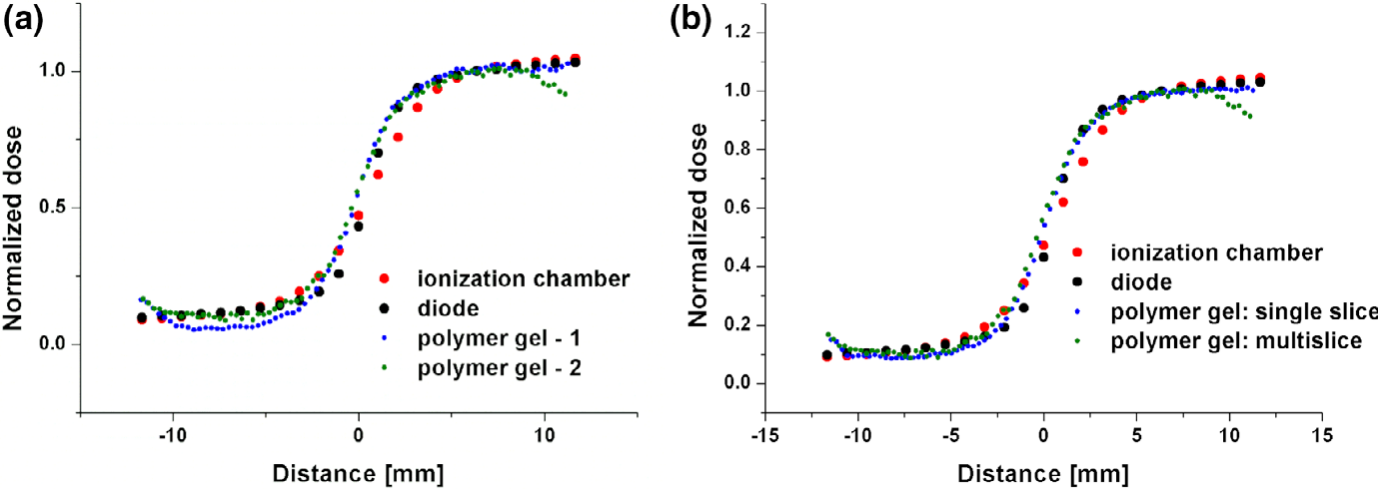
(a) The relative dose distributions measured in both VIPAR^nd^ phantoms using a multislice technique (comparison 3, measurements at days 3‒4 after irradiation, the central slice). (b) The relative dose distributions measured using a basic multislice (the central slice) and a single slice sequence (comparison 2, measurements at day 3 after irradiation). The results obtained with VIPAR^nd^ are compared with the ionizing chamber and a diode detector data. The uncertainties of the data measured with the single and mutislice sequences are presented in Table S13. [Color figure can be viewed at wileyonlinelibrary.com]

A relatively good interphantom reproducibility was also obtained using a multislice technique [comparison 3, Fig. 10(a)]. The gamma index analysis indicates that the dose distributions measured using the polymer gels are comparable to the data obtained with a silicone diode when assuming 1 mm/4% (or 0.5 mm/6%) criterion for the phantom 1 and 1 mm/2% (or 0.5 mm/10%) limit for the phantom 2. The distances of ±11.5 mm around the beam axis (point 0 mm, see Fig. 10) were taken into account in this analysis for the phantom 1 and from −11.5 to 9.5 mm for the phantom 2. The relative dose distributions measured using a basic multislice sequence (central slice) and a single slice sequence (comparison 2) were shown to be in a close agreement [Fig. 10(b)].

**Fig. 11:**
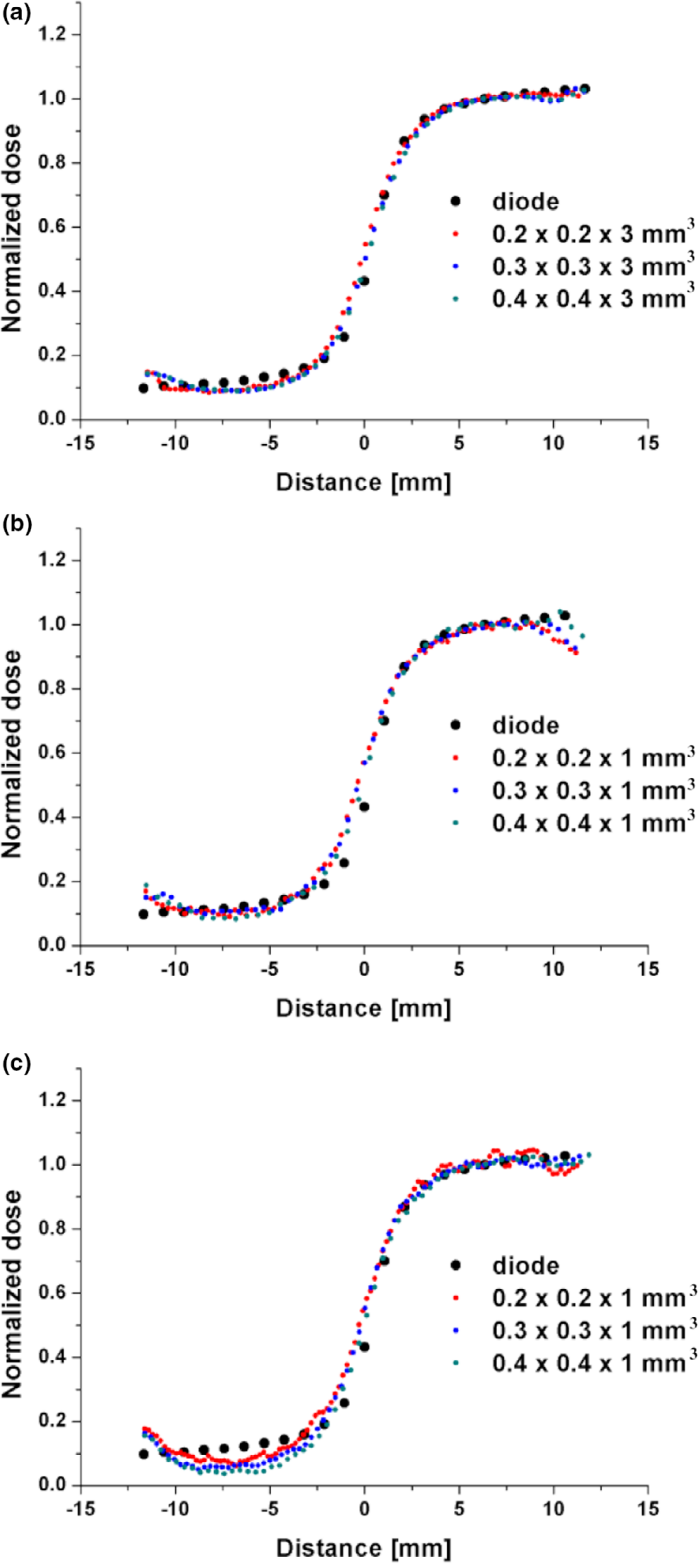
The relative dose distributions measured at varying in‐plane resolutions using: a single slice technique in phantom 2 at day 3 postirradiation (comparison 7) (a), a multislice technique in phantom 2 at day 3 postirradiation (comparison 8) (b), a multislice technique in phantom 1 at day 4 postirradiation (comparison 9) (c). The uncertainties of data measured with the single and mutislice sequences for different voxel dimensions are presented in Tables S14 and S15. [Color figure can be viewed at wileyonlinelibrary.com]

The R2 mapping at different in‐plane resolutions (at days 3‒4 after irradiation) provides similar relative dose profiles both for a single slice (comparison 7) and multislice (comparisons 8 and 9) imaging (Fig. 11).

## 4 Discussion

This work presents the first application of 9.4 T MR microimaging scanner for characterization of dosimetric properties of VIPAR^nd^ gel.

The dose sensitivity of the VIPAR^nd^ gel measured at 9.4 T (0.065 Gy^−1^s^−1^ at day 3 postirradiation) was lower than that reported at 1.5 T (0.088 Gy^−1^s^−1^).^14^ The postirradiation monitoring of the R2‒dose curves confirmed an increase in R2_0_ as a function of time, which is in accordance with other studies.^24^ This effect could be explained by the changes in the conformation of the gelatin triple helices and a gradual strengthening of the interaction within the gelatin chains. Although the relations between the basic acquisition parameters (number of signal acquisitions, voxel size) and a signal to noise ratio in a standard T1‐weighted and a T2‐weighted imaging are well known, their effect on the dose resolution determined from the multiecho sequences is not trivial and deserves experimental investigation. Additionally, a higher gradient strength used in the high field MR microimaging systems leads to a more pronounced diffusion weighting of the signal than in clinical systems. This effect manifests itself as a deviation of the measured R2 from the spectroscopically determined value as a function of voxel volume.^25^ Our results confirm that the increases of the pixel size or in the slice thickness lead to a decrease in the offset of the R2‒dose relation.

The dose resolution of VIPAR^nd^ at 9.4 T for a single slice sequence (0.2 × 0.2 × 3 mm^3^) approaches 0.3 Gy (at *P* = 95%) for four signal averages. Further averaging does not lead to a considerable improvement of this parameter. Interestingly, a four‐fold increase in the voxel volume (obtained by a change of in‐plane resolution from 0.2 × 0.2 mm^2^ to 0.4 × 0.4 mm^2^, 1 signal average) resulted in only a slight improvement of dose resolution (change from 0.4 Gy to 0.3 Gy) at 10 Gy. When taking a multislice sequence (0.2 × 0.2 × 1 mm^3^, 9 slices) into consideration, the dose resolution obtained for 12 signal averages was about 0.5 Gy (at *P* = 95%) for the evaluated dose range.

It is difficult to directly compare the dose resolutions obtained using *N*‐vinylpyrrolidone based gels at various MRI systems due to the differences in the gel dosimeter compositions, imaging time periods and a huge variation in the acquisition parameters. However, it is illustrative that Kipouros et al. reported a dose resolution of 0.5 Gy at *P* = 52% (corresponding to 1.4 Gy at *P* = 95%) at a dose of 10 Gy using 3D acquisition technique at 1.5 T for a voxel size of 0.75 × 0.75 × 1.5 mm^3^.^26^ This value is comparable to the dose resolution obtained by us using a multislice technique (0.2 × 0.2 × 1 mm^3^, 9 slices, 1 average) for 20‐fold lower voxel volume. Of note, a dose sensitivity of the gel studied by Kipouros et al. (0.073 Gy^−1^ s^−1^) was similar to the value obtained in our work. Papadakis et al. also reported the dose resolution at 10 Gy to be ca. 1.3 Gy (using a 2D multislice technique at 1.5 T), albeit for a considerably larger voxel size of 1 × 1 × 5 mm^3^.^27^ The volume of this voxel is 120‐fold higher than the volume obtained in a mutislice sequence in our study. Even taking into account that the gel dosimeter used by Papadakis et al.^27^ was characterized by a lower dose sensitivity (0.043 Gy^−1^s^−1^) than the VIPAR^nd^ gel dosimeter, the gain obtained from using our methodology for the dose read‐out is evident. A higher field strength and proximity of the detection coil to the sample are mainly responsible for this effect. However, direct comparison of results obtained using clinical (1.5T and 3T) and 9.4 T scanner within one experimental procedure is required to fully assess the benefits of ultra‐high B0 field microimaging for high spatial resolution measurements of dose distributions in radiotherapy.

The *N*‐vinylpyrrolidone based gels were imaged using a 32‒echo sequence in the abovementioned studies. A large improvement of the dose resolution obtained by increasing the number of echoes from 32 to 90 under a fixed total echo time was experimentally shown in our study. This result is in accordance with Baldock et al. suggesting that the number of echoes should be as high as possible within an optimal total echo time.^28^ Interestingly, the dose resolution obtained for a highly sensitive variant of *N*‐vinylpyrrolidone based gels (iVIPET, α = 0.238 Gy^−1^s^−1^) imaged at only two echo times at 1.5 T^4^ was similar to the dose resolution of VIPAR^nd^ imaged using a single slice 90‒echo technique (0.2 × 0.2 × 3 mm^3^, 1 average) as in our work (for 16‐fold lower voxel volume).

The multiecho sequences with unlimited number of echoes are usually more available at high field microimaging research systems than on clinical scanners. One of the drawbacks of the system used in this work is a small bore size (30 mm diameter) restricting the dimensions of the gel phantom. However, in recent years there is an increasing demand for dose distribution imaging in small volumes and high field microimaging systems could be effectively exploited for this purpose. Moreover, there are also high field MR systems available with the bore sizes of up to 30 cm.

*N*‐vinylpyrrolidone based gels were successfully applied for the small field dosimetry at clinical scanners.^10^, ^11^ The importance of appropriate detector size in these applications was shown by Pappas et al.^10^, ^11^ The penumbra widths of the narrow (5 and 10 mm) beams determined with the polymer gel dosimeters were lower than those obtained using film, pinpoint or ionization chamber,^11^ while a good agreement between the gel derived data and the dose profiles measured with a diamond dosimeter and a silicon‐diode array was reported elsewhere.^10^ The influence of a volume averaging effect caused by the finite detector dimensions on the dose profile of 5 mm photon stereotactic beam was studied using PAGAT gel.^29^ The penumbra width of this beam was shown to be linearly dependent on the detector size falling into the range of 1.5‒5.5 mm, while the size of 0.5 mm was sufficient to adequately measure the true penumbra width of 1.7 mm.

The error related to the nonzero detector size is dependent on the actual dose gradient. Since there is not a “gold standard” dosimeter for small beams, in our preliminary work we decided to check the performance of the VIPAR^nd^–9.4 T MR microimaging system for characterization of the half‐field beam penumbra (10 × 10 cm^2^). The 80‒20% penumbra width of the evaluated dose distribution was equal to 3.7 mm as determined with a diode detector, 3.9 ± 0.15 mm — with a polymer gel and 4.19 ± 0.09 mm — with a radiochromic film. A general good agreement between these detectors is shown. A spatial resolution ~1 mm offered by the diode detector was assumed to be sufficiently small to produce true penumbral width for a half‐beam gradient area investigated in our study. Similarly, Fox et al. used the Edge diode detector (Sun Nuclear Incorporated, USA) characterized by a sensitive volume diameter of 0.8 mm for the measurements of the 10 × 10 cm^2^ profiles.^30^ The close agreement between the dose distributions obtained with the diode and gel detectors at day 3 postirradiation indicates that the spatial resolution offered by them is adequate to avoid the volume averaging for the evaluated dose gradient. Although the spatial resolution of 0.2–0.4 mm was not required in our work, a good agreement between the gel and diode at day 3 after irradiation encourages to extend the investigations to small radiation fields. As expected, the overestimation of the penumbra width measured with a Semiflex 3D ionization chamber was observed. Because of its finite size (sensitive volume diameter of 4.8 mm) that introduces the volume averaging effects and the presence of air within its active volume, this chamber is not a suitable detector to characterize the evaluated profile penumbra region.

Radiochromic film dosimetry was considered as another tool to obtain reliable data for assessing the suitability of gel dosimetry for area of dose gradient characterization.^31^ This technique offers a high spatial resolution, tissue equivalence and weak energy dependence when used in high energy photon beams. The commonly used scan resolution in small field dosimetry application is from 150 to 75 dpi (0.17 ‐ 0.35 mm/pixel) (reviewed in^32^). These values are comparable to the pixel dimensions used in our work. The mentioned films are now widely used for 2D dose distribution evaluation in conformal radiotherapy where the fractional doses are about 2 Gy. However, they are characterized by a limited dose resolution for the doses typically used in stereotactic radiosurgery (5‒20 Gy). Marroquin et al. found a mean dose resolution of 2.3% for the dose range from 6 to 35 Gy at a confidence level *P* = 68% (corresponding to 4.5% at *P* = 95%) for a spatial resolution of 72 dpi (0.35 × 0.35 mm).^33^ This value could be directly compared to the dose resolution of about 0.3 Gy at the doses from 8 to 30 Gy [mean dose resolution of 2% at *P* = 95%, see Fig. 4(b)] obtained for a single slice technique (0.4 × 0.4 × 3 mm^3^, 1 average) in our work. A slight broadening of the penumbra width measured with EBT3 film as compared to the diode detector was reported by Larraga‐Gutierrez et al.^34^ Such behavior of the film dosimetric system was attributed to the blurring effect caused by the scanner.^35^

The spatial and temporal integrity of the dose distributions in two verification phantoms was monitored over 10 days after irradiation. This monitoring revealed a good interphantom reproducibility of the technique. However, a large underestimation in the low dose region was detected starting from day 5 after irradiation. Some problems with dosimetric precision and accuracy of the *N*‐vinylpyrrolidone based polymer gels in the low dose regions have been previously reported,^26^, ^36^ however, for the first time for the VIPAR dosimeter. These problems were mainly caused by a nonlinearity of the R2‐dose response and the reduced dose resolution for the low doses of ionizing radiation. However, no clear deviations from the R2‒dose linearity and a quite uniform dose resolution across the evaluated dose range was obtained using four signal averages in the single slice sequence in our study. The observations related to the stability of VIPAR^nd^ after irradiation (R2_0_) might be somehow related also to the unknown transportation temperature history. Also, we can speculate after the experiment performed that oxygen leakage through the thin walls of the poly(methyl methacrylate) made containers is very likely, despite the containers were manufactured with due diligence. This may affect the gel dosimeter chemical structure for longer storage time as well as its dose response. Some solution to this problem might be storing the containers with dosimeters submerged in water or in bags containing oxygen scavengers, which however requires further studies.

## 5 Conclusions

This work presents the first application of 9.4 T MR microimaging scanner equipped with a radiofrequency coil with an inner diameter of 30 mm for characterization of the dosimetric properties of the Vipar^nd^ polymer gel. Using the multiecho (90 × 7 ms) single slice (0.2 × 0.2 × 3 mm^3^) and multislice (0.2 × 0.2 × 1 mm^3^, 9 slices) sequences it was possible to obtain a dose resolution at 10 Gy (at *P* = 95%) of 0.42 Gy and 2 Gy, respectively, for one signal acquisition (measurement time: 15 min). These values could be improved to 0.3 Gy and 1 Gy, respectively, after the reduction in the in‐plane spatial resolution to 0.4 × 0.4 mm^2^ (measurement time: 7.5 min). A good agreement between the dose distributions measured in the phantoms irradiated in the half beam penumbra area using the Vipar^nd^ gel and diode detectors was obtained at days 3–4 postirradiation as indicated by the gamma index analysis assuming 1 mm/5% criterion. The presented dose read out procedure could be useful for the high spatial resolution measurements of dose distributions in modern radiotherapy techniques utilizing small irradiation fields (<3 cm), large doses per fraction and wide dose range (5‒25 Gy). However, further studies are required to improve a dosimetry accuracy at low doses of ionizing radiation and to improve temporal stability of the gel derived dose distributions.

## Conflict of Interest

The authors have no conflict to disclose.

## Supporting information


**Supplementary Material** **.** The signal to noise ratio (SNR) of the spin echo images measured using a multiecho (7 ms × 90) sequence.

**Supplementary Material** **.** Relationship between the relative R2 uncertainty and the number of echoes included in a mono‐exponential fitting.

**Table S4** **.** The temporal evolution of the R2‐dose relation measured using a basic single slice sequence (0.2 × 0.2 × 3 mm^3^, NSA = 1) over 14 days after irradiation of the VIPAR^nd^ samples. The mean R2, mean standard R2 uncertainty σ_R2 _in the circular region of interest positioned in the phantom center and the relative standard uncertainty of R2 computed as (σ_R2_/R2)*100% are provided.

**Table S5** **.** The temporal evolution of the R2‐dose relation measured using a basic multislice sequence (0.2 × 0.2 × 1 mm^3^, NSA = 1) over 14 days after irradiation of the VIPAR^nd^ samples. The mean R2, mean R2 standard uncertainty σ_R2 _in the circular region of interest positioned in the phantom center and the relative standard uncertainty of R2 computed as (σ_R2_/R2)*100 % are provided. Additionally. the R2‐dose relation obtained for a single slice sequence (0.2 × 0.2 × 1 mm^3^, NSA = 1) at day 3 postirradiation is shown.

**Table S6** **.** The R2‐dose relations for different in‐plane resolutions and slice thicknesses for a single slice sequence (day 3 after irradiation, NSA = 1). The mean R2, mean R2 standard uncertainty σR2 and relative standard uncertainty of R2 computed as (σR2/R2) × 100%.

**Table S7** **.** The R2‐dose relations for different in plane resolutions and slice thicknesses for a multislice sequence (day 4 after irradiation, NSA = 1). The mean R2, mean standard uncertainty σR2 and relative standard uncertainty of R2 computed as (σR2/R2) × 100 % in the circular region of interest positioned in the phantom center are provided.

**Table S8** **.** The standard uncertainties corresponding to the normalized dose profiles through the irradiated‐unirradiated transition zone obtained using a basic single slice microimaging sequence (0.2 × 0.2 × 3 mm^3^, NSA = 4) of two VIPAR^nd^ verification gel vials at day 3 after irradiation [Fig. 9(a)]. A standard uncertainty of the normalized dose was computed based on the R2 standard uncertainty.

**Table S9** **.** The standard uncertainties corresponding to the normalized dose profiles through the irradiated‐unirradiated transition zone obtained using a basic single slice microimaging sequence (0.2 × 0.2 × 3 mm^3^, NSA = 4) of two VIPAR^nd^ verification gel vials at day 5 after irradiation [Fig. 9(b)]. A standard uncertainty of the normalized dose was computed based on the R2 standard uncertainty.

**Table S10** **.** The standard uncertainties corresponding to the normalized dose profiles through the irradiated‐unirradiated transition zone obtained using a basic single slice microimaging sequence (0.2 × 0.2 × 3 mm^3^, NSA = 4) of two VIPAR^nd^ verification gel vials at day 7 after irradiation [Fig. 9(c)]. A standard uncertainty of the normalized dose was computed based on the R2 standard uncertainty.

**Table S11** **.** The standard uncertainties corresponding to the normalized dose profiles through the irradiated‐unirradiated transition zone obtained in phantom 1 using a basic single slice microimaging sequence (0.2 × 0.2 × 3 mm^3^, NSA = 4) of two VIPAR^nd^ verification gel vials at day 10 after irradiation [Fig. 9(d)]. A standard uncertainty of the normalized dose was computed based on the R2 standard uncertainty.

**Table S12** **.** The normalized dose profile (presented in Fig. 9) obtained with the use of film dosimetry. The mean and standard deviations were obtained by averaging 30 adjacent dose profiles.

**Table S13** **.** The standard uncertainty corresponding to the normalized dose distributions in the Vipar^nd^ phantom obtained using the single slice (0.2 × 0.2 × 3 mm^3^, NSA = 4) and multislice (0.2 × 0.2 × 1 mm^3^. NSA = 12) techniques [Fig. 10(b)]. A standard uncertainty of the normalized dose was computed based on the R2 standard uncertainty.

**Table S14** **.** The standard uncertainties corresponding to the normalized dose profiles measured at varying in‐plane resolutions using a single slice technique in phantom 2 at day 3 postirradiation [Fig. 11(a)]. A standard uncertainty of the normalized dose was computed based on the R2 standard uncertainty.

**Table S15** **.** The standard uncertainties corresponding to the normalized dose profiles measured at varying in‐plane resolutions using a multislice technique in phantom 1 at day 4 postirradiation [Fig. 11(c)]. A standard uncertainty of the normalized dose was computed based on the R2 standard uncertainty.
