# Supplementary material for: Application of high field magnetic resonance microimaging in polymer gel dosimetry
Source: Med Phys. 2020 May 15;47(8):3600–13. doi: 10.1002/mp.14186 (PMC7496647; doi:10.1002/mp.14186)
Supplement: Supplementary file 1 — Supplementary Material . The signal to noise ratio (SNR) of the spin echo images measured using a multiecho (7 ms × 90) sequence. [file MP-47-3600-s001.doc]

**The signal to noise ratio (SNR) of the spin echo images measured using a multiecho**

**(7 ms x 90) sequence**

The SNR ratio was computed according to the equation (Watanabe et al., 2011; Gudbjartsson et al., 1995):


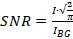


Where I – mean signal intensity in the analyzed image region,

I_BG_ – mean signal intensity in the region of interest located in the background.

Watanabe Y, Kubo H. A variable echo-number method for estimating R2 in MRI-based polymer gel dosimetry. Medical Physics. 2011;38(2):975-982. doi:10.1118/1.3544659.

Gudbjartsson H, Patz S. The rician distribution of noisy mri data. Magnetic Resonance in Medicine. 1995;34(6):910-914. doi:10.1002/mrm.1910340618.

Table S1 presents the signal to noise ratios computed for the spin echo images of the calibration vials (irradiated to the doses of 1.5 Gy, 10 Gy and 20 Gy) acquired using a basic single slice sequence (0.2 x 0.2 x 3 mm^3^, 7 ms x 90, NSA = 1) at day 3 after irradiation. The images acquired at echo times of 35 ms and 640 ms were evaluated.

| Dose [Gy] | TE [ms] | SNR |
| --- | --- | --- |
| 1.5 | 35 | 229.1 |
|  | 640 | 36.1 |
| 10 | 35 | 263.8 |
|  | 640 | 21.1 |
| 20 | 35 | 258.3 |
|  | 640 | 15.2 |

**Table S1. The signal to noise ratios (SNRs) computed for the images acquired at TE = 35 ms and 640 ms using a basic single slice sequence (0.2 x 0.2 x 3 mm^3^, NSA = 1) at day 3 after irradiation.**

Table S2 presents the signal to noise ratios computed for the spin echo images of the calibration vials (irradiated to the doses of 1.5 Gy, 10 Gy and 20 Gy) acquired using a basic multislice (0.2 x 0.2 x 1 mm^3^, 7 ms x 90, NSA = 1) at day 4 after irradiation. The images acquired at echo times of 35 ms and 640 ms were evaluated.

| Dose [Gy] | TE [ms] | SNR |
| --- | --- | --- |
| 1.5 | 35 | 63.3 |
|  | 640 | 5.4 |
| 10 | 35 | 65.0 |
|  | 640 | 3.4 |
| 20 | 35 | 62.1 |
|  | 640 | 2.3 |

**Table S2. The signal to noise ratios (SNR) computed for the images acquired at TE = 35 ms and 640 ms using a basic multislice multiecho sequence (0.2 x 0.2 x 1 mm^3^, NSA = 1) at day 4 after irradiation.**

The representative spin echo images of the verification phantoms measured at TE = 35 and 640 ms using a basic single slice (0.2 x 0.2 x 3 mm^3^, NSA = 4) and a multislice technique (0.2 x 0.2 x 1 mm^3^, NSA = 12) are also shown in Figures S1 and S2. The signal to noise ratios obtained for these images are shown in Table S3.


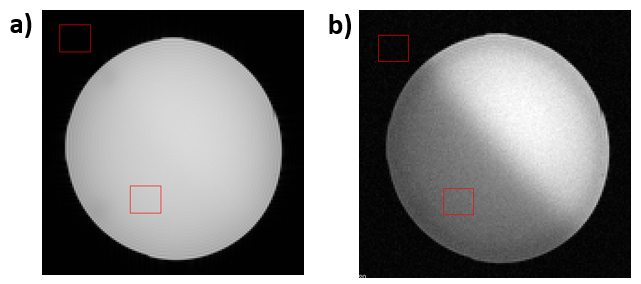


**Figure S1. The spin echo images of the verification phantom measured at TE = 35 (a) and 640 ms (b) using a basic single slice sequence (0.2 x 0.2 x 3 mm^3^, NSA = 4) at day 3 after irradiation. The mean signal intensities in the regions of interest located in the phantom and in the background were used for SNR computation.**


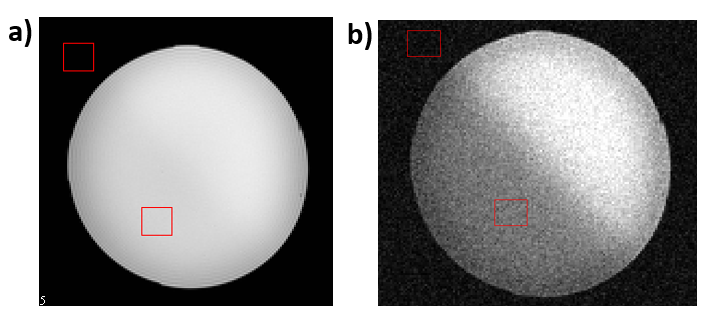


**Figure S2. The spin echo images of the verification phantom measured at TE = 35 (a) and 640 ms (a) using a basic multi - slice sequence (0.2 x 0.2 x 1 mm^3^, NSA = 12) at day 4 after irradiation. The mean signal intensities in the regions of interest located in the phantom and in the background were used for SNR computation.**

| Sequence | TE [ms] | SNR |
| --- | --- | --- |
| basic single slice  0.2 x 0.2 x 3mm^3^, NSA = 4 | 35 | 451 |
|  | 640 | 13.5 |
| basic multislice  0.2 x 0.2 x 1mm^3^, NSA = 12 | 35 | 216.5 |
|  | 640 | 9.5 |

**Table S3. The signal to noise ratios (SNR) computed for the images of the verification phantoms acquired at TE = 35 ms and 640 ms using the basic single (0.2 x 0.2 x 3 mm^3^, NSA = 4) and multislice multi-echo sequences (0.2 x 0.2 x 1 mm^3^, NSA = 12) at days 3- 4 after irradiation.**
