# Supplementary material for: Application of high field magnetic resonance microimaging in polymer gel dosimetry
Source: Med Phys. 2020 May 15;47(8):3600–13. doi: 10.1002/mp.14186 (PMC7496647; doi:10.1002/mp.14186)
Supplement: Supplementary file 2 — Supplementary Material . Relationship between the relative R2 uncertainty and the number of echoes included in a mono‐exponential fitting. [file MP-47-3600-s002.doc]

**Relationship between the relative R2 uncertainty and the number of echoes included in a mono-exponential fitting**

The analysis was performed for:

- the images of the calibration vials acquired at day 3 after irradiation (single slice technique, spatial resolution: 0.2 – 0.4 x 0.2 – 0.4 x 3 mm^3^, 7 ms x 90, NSA = 1)

- the images of the calibration vials acquired at day 4 after irradiation (multi-slice technique, spatial resolution: 0.2 – 0.4 x 0.2 – 0.4 x 1 mm^3^, 7 ms x 90, NSA = 1)

- the images of the calibration vials acquired at day 14 after irradiation (single and multislice technique, in plane spatial resolution: 0.2 – 0.4 x 0.2 – 0.4 mm^2^, slice thickness of 3 mm for a single slice sequence and 1 mm for a multislice sequence, 7 ms x 90, NSA = 1).

The relative R2 uncertainty was computed as (σ_R2_/R2)*100%, where σ_R2_ denotes mean standard uncertainty in the circular region of interest positioned in the phantom center and R2 denotes mean R2 in this region of interest.

The effect of discarding a variable number of last echoes on a relative R2 uncertainty is shown in Figures S3-S6. Of note, the echoes falling within the range from 7‒28 ms were excluded from all analyses due to the imperfections of the spin–spin relaxation decay curve.

It is evident that for all analyzed images the lowest relative R2 uncertainty was obtained after inclusion of all measured echoes. Tables S1-S3 (supplementary file 1) show that the images acquired at the echo time of 640 ms are characterized by a relatively high SNR (above 2). Therefore, the inclusion of the images measured at long TE does not lead to an increased fitting error.

**
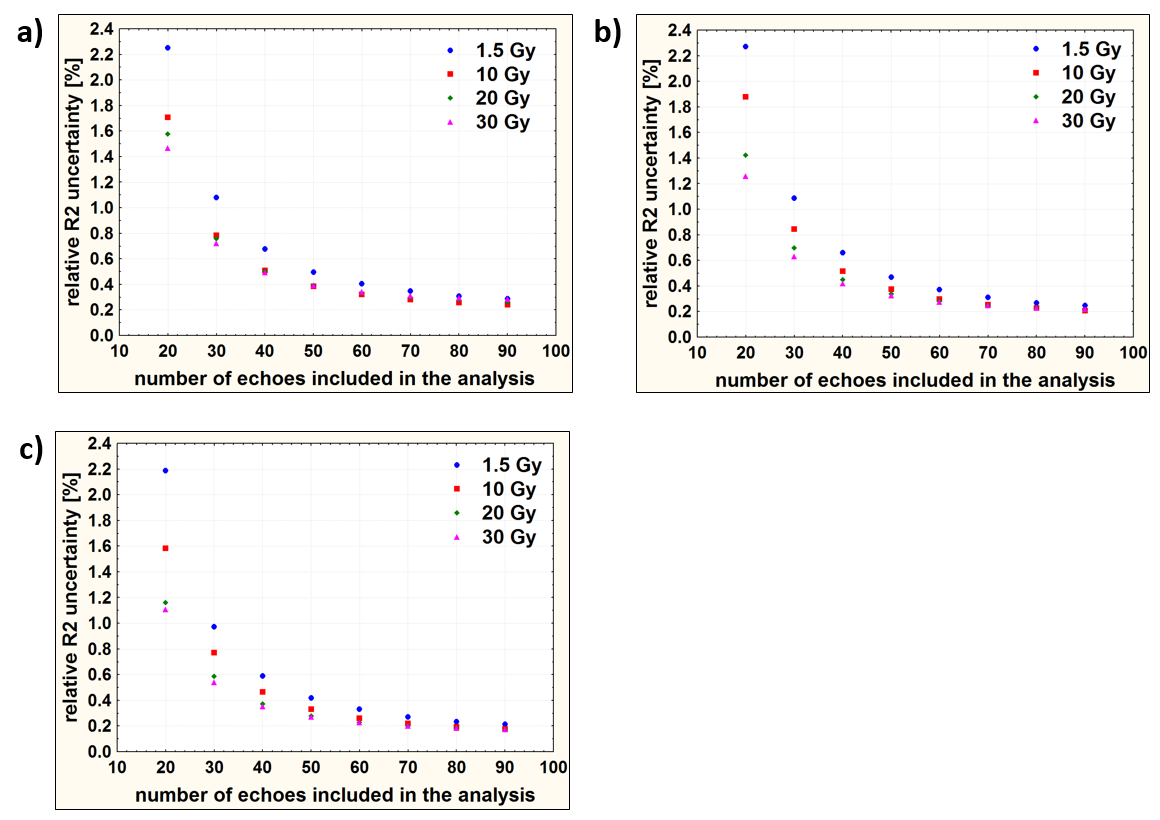
**

**Figure S3. The relationship between the relative R2 uncertainty obtained for the calibration vials (irradiated to 1.5 Gy, 10 Gy, 20 Gy and 30 Gy) and the number of echoes included in the mono-exponential fitting. The images were measured using a single slice technique at day 3 after irradiation at a spatial resolution of 0.2 x 0.2 x 3 mm^3^ (a), 0.3 x 0.3 x 3 mm^3^ (b), 0.4 x 0.4 x 3 mm^3^ (c).**


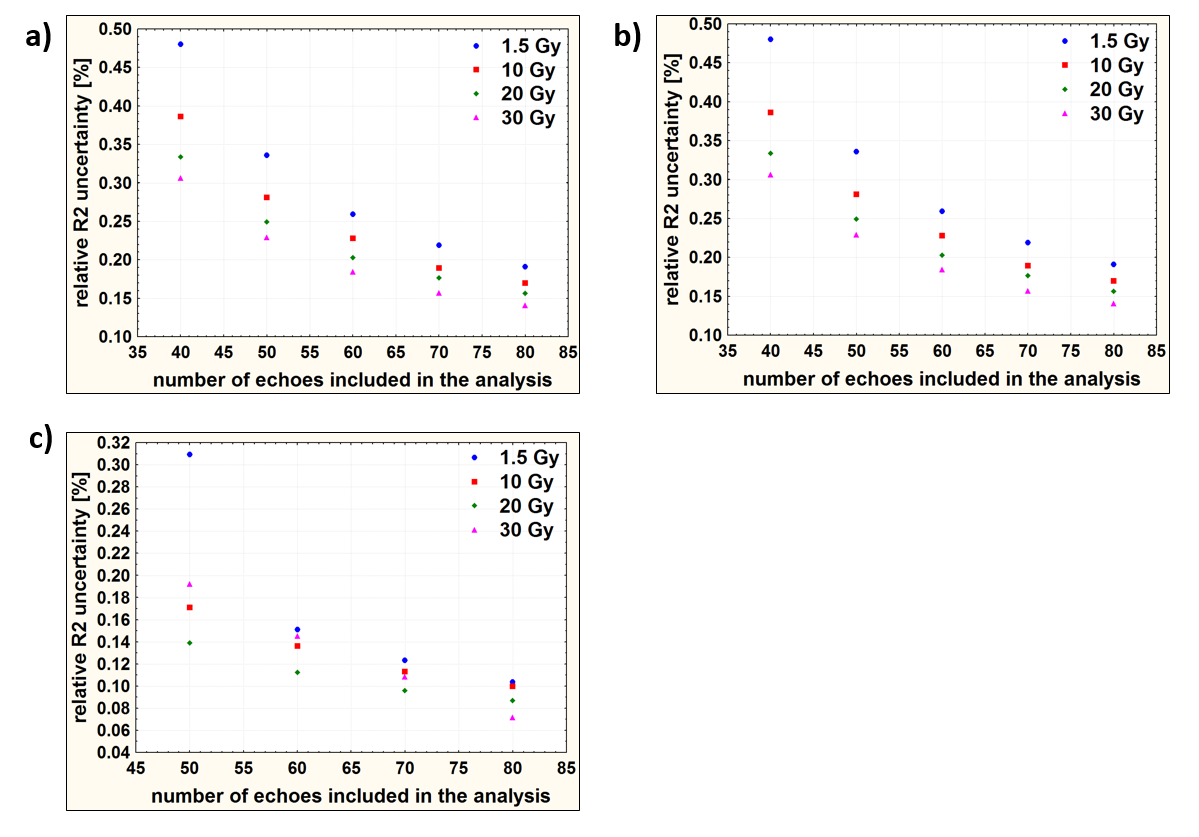


**Figure S4. The relationship between the relative R2 uncertainty obtained for the calibration vials (irradiated to 1.5 Gy, 10 Gy, 20 Gy and 30 Gy) and the number of echoes included in the mono-exponential fitting. The images were measured using a multi - slice technique at day 4 after irradiation at a spatial resolution of 0.2 x 0.2 x 1 mm^3^ (a), 0.3 x 0.3 x 1 mm^3^ (b), 0.4 x 0.4 x 1 mm^3^ (c).**


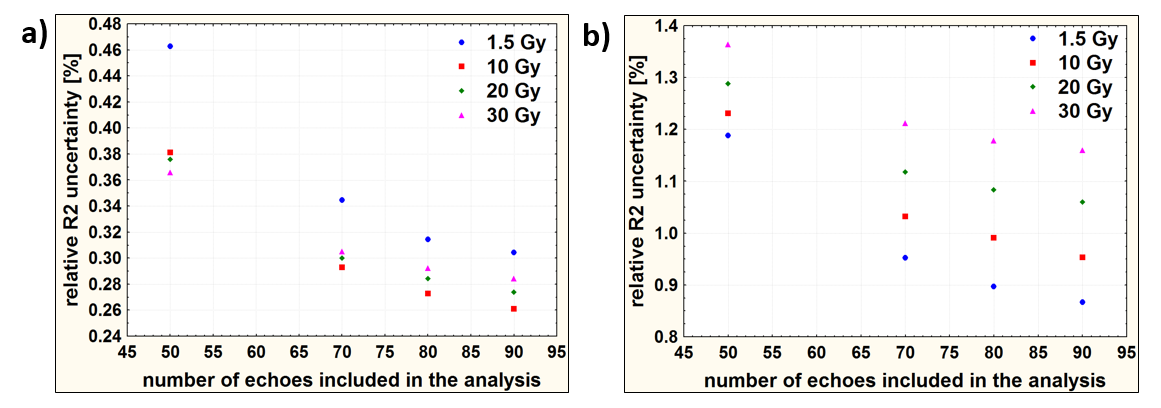


**Figure S5. The relationship between the relative R2 uncertainty obtained for the calibration vials (irradiated to 1.5 Gy, 10 Gy, 20 Gy and 30 Gy) and the number of echoes included in the mono-exponential fitting. The images were measured at day 14 after irradiation using a single slice technique at a spatial resolution of 0.2 x 0.2 x 3 mm^3^ (a) and using a multislice technique at a spatial resolution of of 0.2 x 0.2 x 1 mm^3^ (b).**
