# Supplementary material for: Application of high field magnetic resonance microimaging in polymer gel dosimetry
Source: Med Phys. 2020 May 15;47(8):3600–13. doi: 10.1002/mp.14186 (PMC7496647; doi:10.1002/mp.14186)
Supplement: Supplementary file 4 — Table S5 . The temporal evolution of the R2‐dose relation measured using a basic multislice sequence (0.2 × 0.2 × 1 mm3, NSA = 1) over 14 days after irradiation of the VIPARnd samples. The mean R2, mean R2 standard uncertainty σR2 in the circular region of interest positioned in the phantom center and the relative standard uncertainty of R2 computed as (σR2/R2)*100 % are provided. Additionally. the R2‐dose relation obtained for a single slice sequence (0.2 × 0.2 × 1 mm3, NSA = 1) at day 3 postirradiation is shown. [file MP-47-3600-s004.doc]

|  | Day 4 | | | Day 5 | | | Day 7 | | | Day 10 | | | Day 14 | | | Day 3, 1 slice | | |
| --- | --- | --- | --- | --- | --- | --- | --- | --- | --- | --- | --- | --- | --- | --- | --- | --- | --- | --- |
| Dose [Gy] | R2  [s^-1^] | σ_R2_  [s^-1^] | (σ_R2_/R2)*100 [% ] | R2  [s^-1^] | σ_R2_  [s^-1^] | (σ_R2_/R2)*100 [% ] | R2  [s^-1^] | σ_R2_  [s^-1^] | (σ_R2_/R2)*100 [% ] | R2  [s^-1^] | σ_R2_  [s^-1^] | (σ_R2_/R2)*100 [% ] | R2  [s^-1^] | σ_R2_  [s^-1^] | (σ_R2_/R2)*100 [%] | R2  [s^-1^] | σ_R2_  [s^-1^] | (σ_R2_/R2)*100 [%] |
| 0 | 4.334 | 0.038 | 0.89 | 4.168 | 0.036 | 0.86 | 4.313 | 0.038 | 0.89 | 4.184 | 0.033 | 0.80 | 4.575 | 0.041 | 0.91 | 4.201 | 0.021 | 0.50 |
| 1.5 | 4.260 | 0.038 | 0.90 | 4.212 | 0.034 | 0.81 | 4.372 | 0.036 | 0.82 | 4.407 | 0.038 | 0.86 | 4.663 | 0.040 | 0.87 | 4.190 | 0.021 | 0.49 |
| 3 | 4.396 | 0.037 | 0.85 | 4.388 | 0.036 | 0.82 | 4.537 | 0.040 | 0.89 | 4.414 | 0.036 | 0.81 | 4.807 | 0.041 | 0.86 | 4.403 | 0.022 | 0.50 |
| 5 | 4.594 | 0.038 | 0.83 | 4.558 | 0.041 | 0.90 | 4.678 | 0.041 | 0.87 | 4.559 | 0.037 | 0.82 | 5.084 | 0.047 | 0.92 | 4.443 | 0.021 | 0.48 |
| 8 | 4.787 | 0.040 | 0.84 | 4.803 | 0.046 | 0.95 | 4.967 | 0.044 | 0.88 | 4.948 | 0.043 | 0.87 | 5.283 | 0.048 | 0.90 | 4.748 | 0.024 | 0.50 |
| 10 | 4.958 | 0.046 | 0.94 | 4.908 | 0.044 | 0.89 | 5.100 | 0.046 | 0.91 | 5.050 | 0.042 | 0.83 | 5.535 | 0.053 | 0.97 | 4.804 | 0.023 | 0.49 |
| 14 | 5.222 | 0.047 | 0.89 | 5.252 | 0.051 | 0.96 | 5.416 | 0.055 | 1.02 | 5.401 | 0.049 | 0.90 | 5.766 | 0.055 | 0.95 | 5.186 | 0.027 | 0.52 |
| 20 | 5.592 | 0.056 | 1.00 | 5.560 | 0.052 | 0.94 | 5.754 | 0.056 | 0.98 | 5.821 | 0.053 | 0.91 | 6.194 | 0.066 | 1.06 | 5.425 | 0.028 | 0.52 |
| 25 | 5.850 | 0.059 | 1.02 | 5.902 | 0.060 | 1.01 | 6.074 | 0.065 | 1.07 | 6.197 | 0.062 | 1.00 | 6.490 | 0.065 | 1.01 | 5.829 | 0.032 | 0.55 |
| 30 | 6.136 | 0.064 | 1.04 | 6.182 | 0.068 | 1.10 | 6.343 | 0.068 | 1.07 | 6.481 | 0.072 | 1.11 | 6.747 | 0.078 | 1.16 | 6.090 | 0.033 | 0.55 |

**Table S5. The temporal evolution of the R2‒dose relation measured using a basic multi-slice slice sequence (0.2 x 0.2 x 1 mm^3^, NSA = 1) over 14 days after irradiation of the VIPAR^nd^ samples. The mean R2, mean R2 standard uncertainty σ_R2_ in the circular region of interest positioned in the phantom center and the relative standard uncertainty of R2 computed as (σ_R2_/R2)*100 % are provided. Additionally. the R2‒dose relation obtained for a single slice sequence (0.2 × 0.2 × 1 mm^3^, NSA = 1) at day 3 post-irradiation is shown.**
