# Supplementary material for: Application of high field magnetic resonance microimaging in polymer gel dosimetry
Source: Med Phys. 2020 May 15;47(8):3600–13. doi: 10.1002/mp.14186 (PMC7496647; doi:10.1002/mp.14186)
Supplement: Supplementary file 5 — Table S6 . The R2‐dose relations for different in‐plane resolutions and slice thicknesses for a single slice sequence (day 3 after irradiation, NSA = 1). The mean R2, mean R2 standard uncertainty σR2 and relative standard uncertainty of R2 computed as (σR2/R2) × 100%. [file MP-47-3600-s005.doc]

|  | 0.2 x 0.2 x 3 mm^3^ | | | 0.3 x 0.3 x 3 mm^3^ | | | 0.4 x 0.4 x 3 mm^3^ | | | 0.2 x 0.2 x 1 mm^3^ | | | 0.2 x 0.2 x 2 mm^3^ | | |
| --- | --- | --- | --- | --- | --- | --- | --- | --- | --- | --- | --- | --- | --- | --- | --- |
| Dose [Gy] | R2  [s^-1^] | σ_R2_  [s^-1^] | (σ_R2_/R2)*100 [%] | R2  [s^-1^] | σ_R2_  [s^-1^] | (σ_R2_/R2)*100 [%] | R2  [s^-1^] | σ_R2_  [s^-1^] | (σ_R2_/R2)*100 [%] | R2  [s^-1^] | σ_R2_  [s^-1^] | (σ_R2_/R2)*100 [%] | R2  [s^-1^] | σ_R2_  [s^-1^] | (σ_R2_/R2)*100 [%] |
| 0 | 3.628 | 0.011 | 0.30 | 3.159 | 0.008 | 0.24 | 3.159 | 0.008 | 0.24 | 4.201 | 0.021 | 0.50 |  |  |  |
| 1.5 | 3.604 | 0.010 | 0.29 | 3.152 | 0.007 | 0.21 | 3.152 | 0.007 | 0.21 | 4.190 | 0.021 | 0.49 | 3.665 | 0.011 | 0.30 |
| 3 | 3.798 | 0.010 | 0.26 | 3.312 | 0.008 | 0.23 | 3.312 | 0.008 | 0.23 | 4.403 | 0.022 | 0.50 |  |  |  |
| 5 | 3.854 | 0.009 | 0.24 | 3.400 | 0.007 | 0.19 | 3.400 | 0.007 | 0.19 | 4.443 | 0.021 | 0.48 | 3.917 | 0.012 | 0.30 |
| 8 | 4.168 | 0.011 | 0.25 | 3.694 | 0.007 | 0.18 | 3.694 | 0.007 | 0.18 | 4.748 | 0.024 | 0.50 |  |  |  |
| 10 | 4.246 | 0.010 | 0.24 | 3.805 | 0.007 | 0.18 | 3.805 | 0.007 | 0.18 | 4.804 | 0.023 | 0.49 | 4.316 | 0.013 | 0.30 |
| 14 | 4.616 | 0.012 | 0.26 | 4.147 | 0.007 | 0.17 | 4.147 | 0.007 | 0.17 | 5.186 | 0.027 | 0.52 |  |  |  |
| 20 | 4.900 | 0.013 | 0.26 | 4.479 | 0.008 | 0.17 | 4.479 | 0.008 | 0.17 | 5.425 | 0.028 | 0.52 | 4.979 | 0.015 | 0.31 |
| 25 | 5.297 | 0.014 | 0.26 | 4.858 | 0.009 | 0.18 | 4.858 | 0.009 | 0.18 | 5.829 | 0.032 | 0.55 |  |  |  |
| 30 | 5.564 | 0.016 | 0.28 | 5.133 | 0.009 | 0.17 | 5.133 | 0.009 | 0.17 | 6.090 | 0.033 | 0.55 |  |  |  |

**Table S6. The R2-dose relations for different in-plane resolutions and slice thicknesses for a single slice sequence (day 3 after irradiation, NSA = 1). The mean R2, mean R2 standard uncertainty σ_R2_ and relative standard uncertainty of R2 computed as (σ_R2_/R2)*100 % in the circular region of interest positioned in the phantom center are provided.**
