# Supplementary material for: Application of high field magnetic resonance microimaging in polymer gel dosimetry
Source: Med Phys. 2020 May 15;47(8):3600–13. doi: 10.1002/mp.14186 (PMC7496647; doi:10.1002/mp.14186)
Supplement: Supplementary file 6 — Table S7 . The R2‐dose relations for different in plane resolutions and slice thicknesses for a multislice sequence (day 4 after irradiation, NSA = 1). The mean R2, mean standard uncertainty σR2 and relative standard uncertainty of R2 computed as (σR2/R2) × 100 % in the circular region of interest positioned in the phantom center are provided. [file MP-47-3600-s006.doc]

|  | 0.2 x 0.2 x 1 mm^3^  9 slices | | | 0.3 x 0.3 x 1 mm^3^  9 slices | | | 0.4 x 0.4 x 1 mm^3^  9 slices | | | 0.2 x 0.2 x 2 mm^3^  5 slices | | | 0.3 x 0.3 x 2 mm^3^  5 slices | | | 0.4 x 0.4 x 2 mm^3^  5 slices | | | 0.2 x 0.2 x 3 mm^3^  3 slices | | |
| --- | --- | --- | --- | --- | --- | --- | --- | --- | --- | --- | --- | --- | --- | --- | --- | --- | --- | --- | --- | --- | --- |
| Dose [Gy] | R2  [s^-1^] | σ_R2_  [s^-1^] | (σ_R2_/R2)*  100  [%] | R2  [s^-1^] | σ_R2_  [s^-1^] | (σ_R2_/R2)*  100  [%] | R2  [s^-1^] | σ_R2_  [s^-1^] | (σ_R2_/R2)*  100  [%] | R2  [s^-1^] | σ_R2_  [s^-1^] | (σ_R2_/R2)*  100  [%] | R2  [s^-1^] | σ_R2_  [s^-1^] | (σ_R2_/R2)*  100  [%] | R2  [s^-1^] | σ_R2_  [s^-1^] | (σ_R2_/R2)*  100  [%] | R2  [s^-1^] | σ_R2_  [s^-1^] | (σ_R2_/R2)*  100  [%] |
| 1.5 | 4.260 | 0.038 | 0.90 | 3.989 | 0.025 | 0.63 | 3.898 | 0.019 | 0.50 | 3.889 | 0.019 | 0.49 | 3.547 | 0.013 | 0.37 | 3.423 | 0.011 | 0.32 | 3.779 | 0.015 | 0.40 |
| 3 | 4.396 | 0.037 | 0.85 | 4.087 | 0.023 | 0.57 | 3.980 | 0.018 | 0.46 |  |  |  |  |  |  |  |  |  |  |  |  |
| 5 | 4.594 | 0.038 | 0.83 | 4.306 | 0.024 | 0.57 | 4.210 | 0.020 | 0.47 | 4.197 | 0.019 | 0.46 | 3.851 | 0.012 | 0.32 | 3.723 | 0.011 | 0.29 | 4.080 | 0.015 | 0.36 |
| 8 | 4.787 | 0.040 | 0.85 | 4.481 | 0.026 | 0.57 | 4.369 | 0.020 | 0.47 |  |  |  |  |  |  |  |  |  |  |  |  |
| 10 | 4.958 | 0.046 | 0.94 | 4.661 | 0.029 | 0.63 | 4.559 | 0.022 | 0.49 | 4.540 | 0.021 | 0.47 | 4.187 | 0.014 | 0.34 | 4.059 | 0.010 | 0.26 | 4.413 | 0.016 | 0.35 |
| 14 | 5.222 | 0.047 | 0.89 | 4.912 | 0.029 | 0.59 | 4.803 | 0.023 | 0.47 |  |  |  |  |  |  |  |  |  |  |  |  |
| 20 | 5.592 | 0.056 | 1.00 | 5.286 | 0.036 | 0.69 | 5.183 | 0.028 | 0.53 | 5.171 | 0.026 | 0.50 | 4.820 | 0.016 | 0.33 | 4.694 | 0.013 | 0.27 | 5.084 | 0.019 | 0.37 |
| 25 | 5.850 | 0.059 | 1.02 | 5.548 | 0.038 | 0.69 | 5.443 | 0.029 | 0.53 |  |  |  |  |  |  |  |  |  |  |  |  |
| 30 | 6.136 | 0.064 | 1.04 | 5.840 | 0.039 | 0.67 | 5.728 | 0.030 | 0.52 |  |  |  |  |  |  |  |  |  |  |  |  |

**Table S7. The R2-dose relations for different in plane resolutions and slice thicknesses for a multislice sequence (day 4 after irradiation, NSA = 1). The mean R2, mean standard uncertainty σ_R2_ and relative standard uncertainty of R2 computed as (σ_R2_/R2)*100 % in the circular region of interest positioned in the phantom center are provided.**
