# Supplementary material for: Application of high field magnetic resonance microimaging in polymer gel dosimetry
Source: Med Phys. 2020 May 15;47(8):3600–13. doi: 10.1002/mp.14186 (PMC7496647; doi:10.1002/mp.14186)
Supplement: Supplementary file 9 — Table S10 . The standard uncertainties corresponding to the normalized dose profiles through the irradiated‐unirradiated transition zone obtained using a basic single slice microimaging sequence (0.2 × 0.2 × 3 mm3, NSA = 4) of two VIPARnd verification gel vials at day 7 after irradiation [Fig. 9(c)]. A standard uncertainty of the normalized dose was computed based on the R2 standard uncertainty. [file MP-47-3600-s009.doc]

|  | Day 7 | |
| --- | --- | --- |
|  | Phantom 1 | Phantom 2 |
| Distance [mm] | Standard uncertainty | Standard uncertainty |
| -10 | 0.006 | 0.005 |
| -9 | 0.006 | 0.006 |
| -8 | 0.007 | 0.006 |
| -7 | 0.007 | 0.006 |
| -6 | 0.006 | 0.006 |
| -5 | 0.007 | 0.006 |
| -4 | 0.006 | 0.006 |
| -3 | 0.006 | 0.006 |
| -2 | 0.006 | 0.006 |
| -1 | 0.005 | 0.006 |
| 0 | 0.005 | 0.006 |
| 1 | 0.006 | 0.006 |
| 2 | 0.006 | 0.007 |
| 3 | 0.007 | 0.007 |
| 4 | 0.007 | 0.007 |
| 5 | 0.007 | 0.007 |
| 6 | 0.007 | 0.007 |
| 7 | 0.007 | 0.007 |
| 8 | 0.007 | 0.007 |
| 9 | 0.007 | 0.007 |
| 10 | 0.007 | 0.007 |

**Table S10. The standard uncertainties corresponding to the normalized dose profiles through the irradiated-unirradiated transition zone obtained using a basic single slice microimaging sequence (0.2 x 0.2 x 3 mm^3^, NSA = 4) of two VIPAR^nd^ verification gel vials at day 7 after irradiation (Figure 9c). A standard uncertainty of the normalized dose was computed based on the R2 standard uncertainty.**
