# Supplementary material for: Application of high field magnetic resonance microimaging in polymer gel dosimetry
Source: Med Phys. 2020 May 15;47(8):3600–13. doi: 10.1002/mp.14186 (PMC7496647; doi:10.1002/mp.14186)
Supplement: Supplementary file 11 — Table S12 . The normalized dose profile (presented in Fig. 9) obtained with the use of film dosimetry. The mean and standard deviations were obtained by averaging 30 adjacent dose profiles. [file MP-47-3600-s011.doc]

| Distance [mm] | Mean  Dose [Gy] | Standard Deviation  [Gy] |
| --- | --- | --- |
| -10.21 | 0.72 | 1.67 |
| -9.50 | 0.75 | 1.61 |
| -8.09 | 0.80 | 1.46 |
| -6.68 | 0.88 | 1.28 |
| -5.27 | 0.98 | 1.46 |
| -3.86 | 1.17 | 2.94 |
| -2.44 | 1.53 | 1.84 |
| -1.03 | 2.43 | 1.15 |
| 0.38 | 4.22 | 3.92 |
| 1.79 | 6.07 | 7.24 |
| 3.20 | 6.85 | 7.20 |
| 4.61 | 7.10 | 5.98 |
| 6.02 | 7.32 | 5.48 |
| 7.43 | 7.44 | 7.05 |
| 8.84 | 7.53 | 8.27 |
| 10.26 | 7.53 | 6.39 |

**Table S12. The normalized dose profile (presented in Figure 9) obtained with the use of film dosimetry.**

**The mean and standard deviations were obtained by averaging 30 adjacent dose profiles.**
