# Supplementary material for: Application of high field magnetic resonance microimaging in polymer gel dosimetry
Source: Med Phys. 2020 May 15;47(8):3600–13. doi: 10.1002/mp.14186 (PMC7496647; doi:10.1002/mp.14186)
Supplement: Supplementary file 12 — Table S13 . The standard uncertainty corresponding to the normalized dose distributions in the Viparnd phantom obtained using the single slice (0.2 × 0.2 × 3 mm3, NSA = 4) and multislice (0.2 × 0.2 × 1 mm3. NSA = 12) techniques [Fig. 10(b)]. A standard uncertainty of the normalized dose was computed based on the R2 standard uncertainty. [file MP-47-3600-s012.doc]

|  | Single Slice  0.2 x 0.2 x 3 mm^3^ | Multislice  0.2 x 0.2 x 1 mm^3^ |
| --- | --- | --- |
| Distance [mm] | Standard R2 uncertainty | Standard R2 uncertainty |
| -10 | 0.005 | 0.011 |
| -9 | 0.006 | 0.011 |
| -8 | 0.006 | 0.012 |
| -7 | 0.005 | 0.011 |
| -6 | 0.005 | 0.010 |
| -5 | 0.005 | 0.013 |
| -4 | 0.005 | 0.011 |
| -3 | 0.005 | 0.012 |
| -2 | 0.005 | 0.012 |
| -1 | 0.006 | 0.011 |
| 0 | 0.006 | 0.013 |
| 1 | 0.006 | 0.013 |
| 2 | 0.006 | 0.014 |
| 3 | 0.006 | 0.014 |
| 4 | 0.006 | 0.013 |
| 5 | 0.006 | 0.014 |
| 6 | 0.006 | 0.014 |
| 7 | 0.007 | 0.012 |
| 8 | 0.007 | 0.013 |
| 9 | 0.007 | 0.012 |
| 10 | 0.007 | 0.012 |

**Table S13. The standard uncertainty corresponding to the normalized dose distributions in the Vipar^nd^ phantom obtained using the single slice (0.2 x 0.2 x 3 mm^3^, NSA = 4) and multi-slice (0.2 x 0.2 x 1 mm^3^. NSA = 12) techniques (Figure 10b). A standard uncertainty of the normalized dose was computed based on the R2 standard uncertainty.**
