# Supplementary material for: Application of high field magnetic resonance microimaging in polymer gel dosimetry
Source: Med Phys. 2020 May 15;47(8):3600–13. doi: 10.1002/mp.14186 (PMC7496647; doi:10.1002/mp.14186)
Supplement: Supplementary file 13 — Table S14 . The standard uncertainties corresponding to the normalized dose profiles measured at varying in‐plane resolutions using a single slice technique in phantom 2 at day 3 postirradiation [Fig. 11(a)]. A standard uncertainty of the normalized dose was computed based on the R2 standard uncertainty. [file MP-47-3600-s013.doc]

| Single slice sequence, Phantom 2 | | | | | | |  |
| --- | --- | --- | --- | --- | --- | --- | --- |
|  | | 0.2 x 0.2 x 3 mm^3^ | | 0.3 x 0.3 x 3 mm^3^ | | 0.4 x 0.4 x 3 mm^3^ | |
| Distance [mm] | Standard R2 uncertainty | | Standard R2 uncertainty | | Standard R2 uncertainty | | |
| -10 | 0.005 | | 0.004 | | 0.004 | | |
| -9 | 0.006 | | 0.004 | | 0.004 | | |
| -8 | 0.006 | | 0.005 | | 0.004 | | |
| -7 | 0.005 | | 0.005 | | 0.004 | | |
| -6 | 0.005 | | 0.005 | | 0.004 | | |
| -5 | 0.005 | | 0.005 | | 0.004 | | |
| -4 | 0.005 | | 0.005 | | 0.004 | | |
| -3 | 0.005 | | 0.004 | | 0.004 | | |
| -2 | 0.005 | | 0.004 | | 0.004 | | |
| -1 | 0.006 | | 0.005 | | 0.003 | | |
| 0 | 0.006 | | 0.004 | | 0.004 | | |
| 1 | 0.006 | | 0.004 | | 0.004 | | |
| 2 | 0.006 | | 0.005 | | 0.005 | | |
| 3 | 0.006 | | 0.005 | | 0.005 | | |
| 4 | 0.006 | | 0.005 | | 0.005 | | |
| 5 | 0.006 | | 0.005 | | 0.005 | | |
| 6 | 0.006 | | 0.006 | | 0.005 | | |
| 7 | 0.007 | | 0.006 | | 0.005 | | |
| 8 | 0.007 | | 0.006 | | 0.005 | | |
| 9 | 0.007 | | 0.006 | | 0.005 | | |
| 10 | 0.007 | | 0.006 | | 0.005 | | |

**Table S14. The standard uncertainties corresponding to the normalized dose profiles measured at varying in-plane resolutions using a single slice technique in phantom 2 at day 3 post-irradiation (Figure 11a). A standard uncertainty of the normalized dose was computed based on the R2 standard uncertainty.**
